# Peroxisomal catalase and plasmalogen biosynthesis protect from oxidative stress in Barth syndrome cardiomyopathy

**DOI:** 10.1007/s00395-026-01197-2

**Published:** 2026-06-30

**Authors:** Elsie Kajese, Malte Hachmann, Katharina J. Ermer, Manuela Erk, Lin Alhasaan, Michael Kohlhaas, Heike Bömmel, Lisa Berberich, Christopher Carlein, Hanna Eberl, Katrin Streckfuß-Bömeke, Leticia Prates Roma, Süleyman Ergün, Christoph Maack, Srikanth Karnati, Jan Dudek

**Affiliations:** 1 Department of Translational Research, Comprehensive Heart Failure Center, University Hospital Würzburg, Am Schwarzenberg 15, Haus A15, 97078 Würzburg, Germany; 2 Institute of Anatomy and Cell Biology, University of Würzburg, Würzburg, Germany; 3 CIPMM - Biophysics Department, Zentrum Für Human- Und Molekularbiologie - Universität Des Saarlandes, 66421 Homburg, Germany; 4 Institute of Pharmacology and Toxicology, University of Würzburg, Würzburg, Germany; 5 Atlas University Research Center (ARC), Atlas University, Istanbul, Turkey; 6 Medical Clinic 1, University Hospital Würzburg, Würzburg, Germany

**Keywords:** Mitochondria, Peroxisomes, Barth syndrome, Catalase, Plasmalogens, Integrated stress response, Apoptosis

## Abstract

Barth Syndrome (BTHS) is an inherited mitochondrial cardiomyopathy caused by variants in the gene encoding TAFAZZIN (*Taz*), a transacylase catalyzing the synthesis of the essential mitochondrial phospholipid cardiolipin (CL). Although defects in *Taz* deteriorate mitochondrial respiration, Ca^2+^-uptake, and redox regulation in cardiac myocytes, we previously observed an unexpected lack of oxidative cardiac damage, despite the development of cardiomyopathy in a BTHS mouse model with *Taz*-knockdown (KD). Furthermore, we revealed that the integrated stress response (ISR) governs metabolic rewiring in *Taz*-KD hearts to compensate for deficient mitochondrial FAO and to support GSH production. Here, we interrogated whether adaptive mechanisms in peroxisomes, which are closely associated with mitochondria and harbor antioxidative enzymes, can also compensate for the mitochondrial defects. We identified alterations in the peroxisomal biogenesis factors PEX14 and PEX19, indicating changes in the peroxisomal proteome in *Taz*-KD vs. WT hearts. While the enzymes of peroxisomal FAO were unchanged, levels of Lon Protease 2 (LONP2) and catalase were elevated in *Taz*-KD hearts. Inhibition or siRNA-mediated knockdown of catalase increased reactive oxygen species (ROS) and blunted the protection of mouse embryonic fibroblasts (MEF) with *Taz*-knockout (KO), but not in WT, from ROS-induced activation of the apoptotic caspase 3. Furthermore, we observed that the increase in plasmalogen synthesis in cardiac *Taz*-KD peroxisomes contributes to the activation of the ISR, since siRNA-mediated knockdown of the key enzyme GNPAT blunted the ISR and thereby increased cellular ROS in *Taz*-KO, but not WT MEFs. In conclusion, peroxisomes facilitate a counterregulatory response to dysfunctional mitochondria by activating a catalase-driven ROS defense and maintaining ISR-mediated metabolic alterations, both of which compensate for mitochondrial dysfunction and oxidative stress. Therefore, the so far poorly investigated mitochondrial-peroxisome crosstalk may represent a novel therapeutic target in an orphan disease with a poor prognosis.

**Supplementary Information:**

The online version contains supplementary material available at https://doi.org/10.1007/s00395-026-01197-2.

## Highlights

- Mitochondrial dysfunction due to a defect in the biogenesis of the phospholipid cardiolipin induces alterations in peroxisomes in a mouse model for Barth syndrome cardiomyopathy.
- Elevated catalase protects from reactive oxygen species-induced apoptosis.
- Plasmalogen biosynthesis in cardiolipin-deficient cells plays a protective role by facilitating the activation of the integrated stress response and preventing apoptosis.

**Supplementary Information:**

The online version contains supplementary material available at https://doi.org/10.1007/s00395-026-01197-2.

## Introduction

Barth syndrome (BTHS) is a mitochondrial disorder characterized by early onset of cardiomyopathy, skeletal muscle myopathy, immune defects, and growth retardation [4]. The pathogenic mutation resides in the X-chromosome-linked gene encoding the mitochondrial transacylase TAFAZZIN (*Taz*; gene: *Tafazzin*), which catalyzes the maturation of the mitochondrial phospholipid cardiolipin (CL) [8]. Loss of *Taz*-function depletes mature forms of CL, alters its acylation pattern, and increases the abundance of the CL precursor monolysocardiolipin [45]. Different mouse models of this disease have been generated. Mice with a (complete) knockout (KO) of *Taz*, show an often lethal phenotype of dilated cardiomyopathy with severe systolic dysfunction [69, 105]. *Taz*-KD mice, in which *Taz* gene expression is systemically reduced by short hairpin-RNA interference, develop a less severe phenotype. These mice have a longer lifespan, milder systolic dysfunction and a lack of left ventricular dilation, but yet a diminished contractile reserve during β-adrenergic stimulation [7]. This phenotype closely resembles the cardiac phenotype of patients with BTHS who survive the initial weeks after birth [15]. Employing the *Taz*-KD mice, but also human induced pluripotent stem cell-derived cardiac myocytes (iPSC-CM) from patients with BTHS, we and others revealed that *Taz*-deficiency disturbs the lipid composition of the inner mitochondrial membrane, affecting mitochondrial morphology, the structural organization of respiratory chain into “supercomplexes” (respirasomes) and thereby, energy conversion [1, 26, 27, 93]. Besides the respiratory chain, also other macromolecular complexes located in the inner mitochondrial membrane are affected by CL depletion, including Krebs cycle enzymes succinate [27] and pyruvate dehydrogenase [67]. *Taz*-deficiency compromises cardiac myocyte substrate metabolism, since mitochondrial uptake and oxidation of fatty acids, the by far dominant energy source of the heart [78], is deteriorated in mouse models and humans with BTHS [10, 14, 61, 63]. This is caused by downregulation of several enzymes involved in fatty acid oxidation (FAO), including carnitine palmitoyltransferase I and II (CPT1 and 2) [14, 61], Coenzyme A (CoA) biosynthesis [63], and very long chain acyl CoA dehydrogenase (VLCAD) in human myocardium from patients with BTHS [12] and *Taz*-KD hearts [63].

A significant finding is the defect in the mitochondrial Ca^2+^ uniporter (MCU) in *Taz*-KD hearts [7, 33]. Mitochondrial Ca^2+^ uptake and the ensuing activation of Krebs cycle is required to adapt energy supply to demand during cardiac workload transitions through regeneration of nicotinamide adenine dinucleotide (NADH) and at the same time, for restoration of the anti-oxidative capacity through regeneration of nicotinamide adenine dinucleotide phosphate (NADPH) [3]. MCU deficiency in BTHS compromises mitochondrial redox regulation, contributing to arrhythmias and diminished contractile reserve during β-adrenergic stimulation [7, 41]. Surprisingly, we and others neither observed increased ROS in murine *Taz*-KD hearts on the level of isolated mitochondria, cardiac myocytes nor whole hearts in vivo [7, 34]. How mitochondrial defects are compensated to prevent overt oxidative damage in *Taz*-KD hearts is still poorly investigated.

We recently revealed that metabolic compensation is provided through rewiring metabolic fluxes towards more glucose- and glutamate uptake. This process is mediated by induction of the ISR, leading to induction of its key transcription factor ATF4 [14, 61]. Importantly, this metabolic switch was also confirmed in human myocardium from patients with BTHS [12]. Besides energetic benefits, resembled by anaplerotic fuelling of glutamate into the Krebs cycle as well ATP deriving from glycolysis and pyruvate oxidation, the aforementioned metabolic switch also boosts the anti-oxidative capacity, since elevated uptake of glucose allows conversion of glycolytic intermediates into serine and glycine to fuel one-carbon (1C) metabolism and glutathione (GSH) biosynthesis [61]. Furthermore, elevated glutamine uptake facilitates its exchange with cystine via the system x_C_^−^ cysteine transporter, providing important building blocks for GSH synthesis [61]. While these processes support the GSH redox buffer, it is currently unknown whether additional compensatory mechanisms are activated to prevent oxidative stress in BTHS cardiomyopathy.

Peroxisomes are ubiquitous organelles that are located in close proximity to mitochondria, with membranous contact sites between both organelles [75], and play an important role in fatty acid metabolism, phospholipid biosynthesis and redox control [56]. They are crucial for the α- and β-oxidation of long- and branched chain fatty acids, and also for the breakdown of toxic lipids and proinflammatory lipid mediators. The close spatial and functional interaction between peroxisomes and mitochondria allows the exchange of short chain fatty acids, acetyl-CoA and reducing equivalents between both organelles [90]. Since peroxisomal FAO provokes generation of H_2_O_2_ in the first oxidation reaction of each oxidative cycle, these organelles require an efficient machinery to detoxify H_2_O_2_ to H_2_O and O_2_, which is accomplished by peroxisomal catalase [29, 57].

The peroxisomal matrix harbors at least 50 different enzymes that need to be imported from the cytosol by the class of peroxin proteins. Proteins containing a peroxisomal targeting signal of type 1 (PTS1) or type 2 (PTS2) are recognized in the cytosol by specific import receptors, PEX5 and PEX7, respectively [39, 87]. The cargo-loaded receptors are directed to a docking complex at the peroxisomal membrane, including the proteins PEX13, PEX14 and PEX17. The association of PEX14 with PEX5 leads to the formation of a transient pore, which functions as a protein-conducting channel [28]. Mice with a deficient peroxisomal import receptor PEX5 have a defect in peroxisomal biogenesis and show also altered mitochondrial morphology, illustrating the interdependence of both organelles. Not only morphology, but also protein levels and enzymatic activity of respiratory chain complex I are reduced, indicating a functional dependency of mitochondria on peroxisomes [6]. Furthermore, we recently revealed that PEX5 deficiency leads to mitochondrial and contractile dysfunction in human iPSC-CM and adult murine cardiac myocytes, resulting in dilated cardiomyopathy in mice in vivo [42]. However, it is currently unresolved whether also vice versa, peroxisomal function is altered by mitochondrial dysfunction.

In energy demanding tissues, like heart and neuronal tissues, a substantial proportion of the phospholipids are plasmalogens (10–30%) [9]. Plasmalogens are important constituents of cellular membranes, and their biosynthesis is initiated in peroxisomes by the glyceronephosphate-O-acyltransferase (GNPAT), which acylates dihydroxyacetone-phosphate. Subsequently, the alkylglycerone-phosphate synthase (AGPS) replaces the acyl group with a fatty alcohol, provided by the fatty acyl-CoA reductase 1 (FAR1) [9]. The final steps of plasmalogen biosynthesis are taking place in the ER [71, 79]. Plasmalogens are signified as ether lipids with the presence of polyunsaturated fatty acids at the sn-2 position (PUFAs) [9]. It has been suggested that plasmalogens are particularly vulnerable to oxidative stress and preferentially oxidized when exposed to ROS or singlet oxygen [66]. However, this view has been discussed controversially [46]. If plasmalogens function as “sacrificial oxidants”, which spare the oxidation of other membrane lipids [92, 110], or rather promote membrane oxidation has not been analyzed in the context of mitochondrial diseases [30]. Importantly, substantial changes in the plasmalogen content have been reported earlier. Studies using nuclear magnetic resonance (NMR) reported on reductions in the overall levels of plasmenylcholine in *Taz*-KD hearts [55]. Using mass-spectrometry, we found changes in the plasmalogen acyl-chain composition as the most prevalent alteration in *Taz*-KD hearts [38].

Taken together, while peroxisomes have important functions in fatty acid metabolism and redox regulation by their close association with mitochondria, their role in mitochondrial diseases and in particular, in BTHS, have hardly been investigated. Therefore, the main aim of this study was to elucidate whether adaptive remodeling of peroxisomes in BTHS cardiomyopathy may contribute to an increased antioxidative capacity in BTHS hearts [7, 69, 105]. Our findings reveal that while no gross alterations in peroxisomal structure and fatty acid metabolism occur, upregulation of catalase expression and a previously unknown GNPAT-dependent activation of the ISR, which governs metabolic rewiring and GSH synthesis [61], converge to boost cellular anti-oxidative capacity in BTHS hearts. These findings provide a deeper insight into the interplay between mitochondria and peroxisomes in an orphaned disease, that despite the recent approval of a CL-targeted therapy (i.e., of elamipretide [88]) still lacks efficient treatment strategies.

## Results

### Peroxisomal alterations in the BTHS mouse heart

**Fig. 1:**
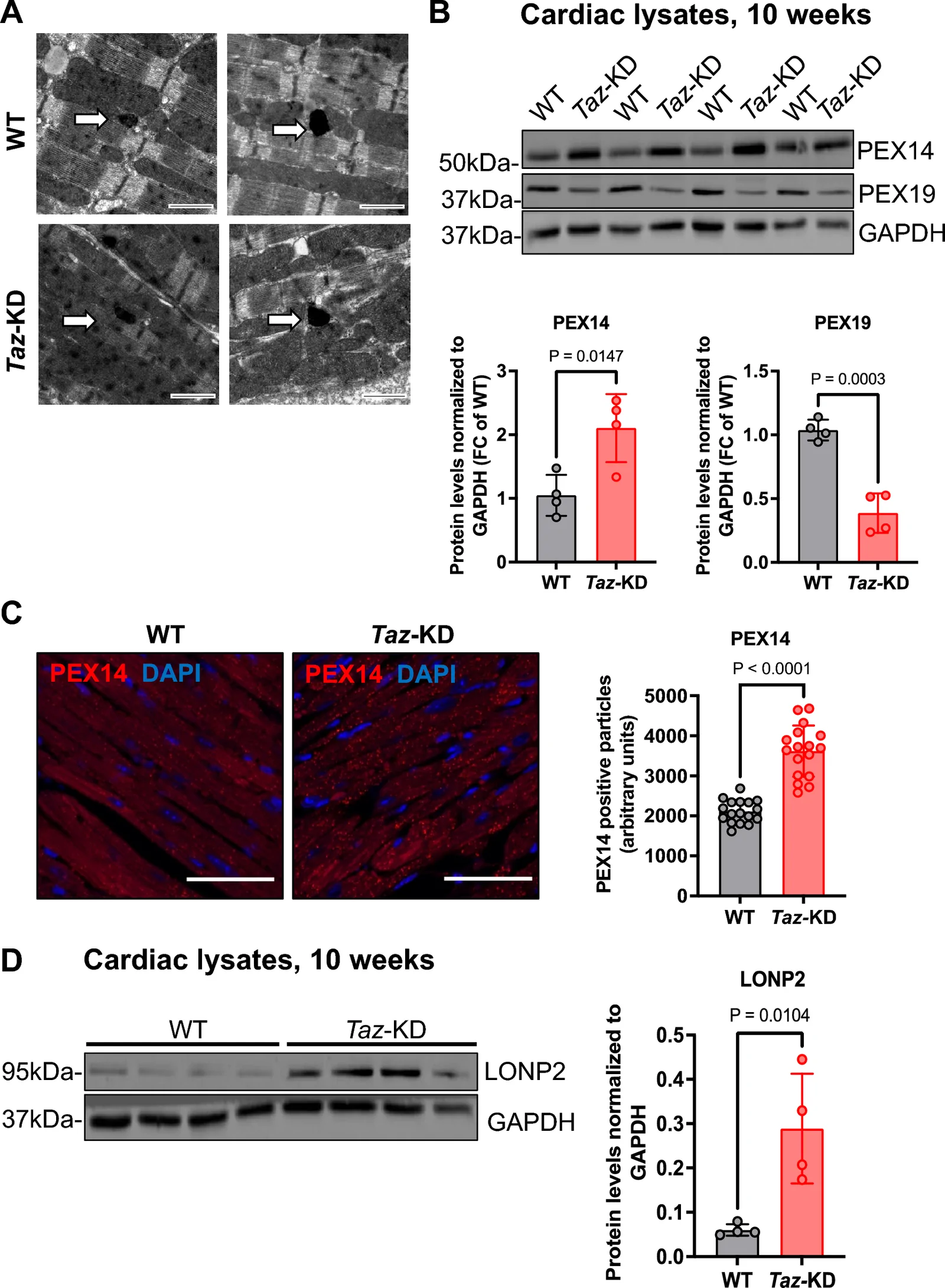
Impact of mitochondrial *Taz-*deficiency on peroxisomal biogenesis. **A** Representative DAB-stained electron micrographs of 50-week-old WT and *Taz*-KD peroxisomes. Scale bar: 1 µm. **B** Western blot of indicated peroxisomal proteins in 10-week-old *Taz*-KD mouse hearts. Quantification of western blots comparing indicated protein levels to the protein expression of GAPDH (values are presented as mean ± SD, n = 4, statistical significance was determined with unpaired Student’s t-test). **C** Immunofluorescence staining of peroxisomal PEX14p (red) and nuclei (DAPI, blue) in 10-week-old WT and *Taz*-KD mouse hearts. Quantification of particles in the right panel (values are presented as mean ± SD, n = 3. Scale bar: 50 µm, statistical significance was determined with unpaired Student’s t-test). **D** Western blot of LONP2 in 10-week-old *Taz*-KD mouse hearts

In the *Taz*-KD mouse model, *Taz* deficiency is induced by shRNA directed against the *Taz* gene [1, 93]. Similar to most adolescent patients with BTHS, *Taz*-KD mice develop mild systolic- but progressive diastolic dysfunction with a blunted β-adrenergic reserve at the age of 8–10 weeks [7, 15, 27, 50]. We confirmed the knockdown of *Taz* gene expression at a juvenile (10 weeks) and adult state (50 weeks, Supplementary Fig. 1A). We analyzed peroxisomes by electron microscopy, employing a diaminobenzidine staining protocol, in which electron-dense products are formed in the presence of peroxisomal catalase. No change in the number and size of peroxisomes or the peroxisomal ultrastructure was observed in *Taz*-KD compared to WT animals (Fig. 1A, Supplementary Fig. 1B). To interrogate peroxisomal biogenesis, we analyzed membrane docking complex proteins PEX14 and PEX19, which are involved in the insertion of peroxisome membrane proteins into peroxisomes [47]. Despite unchanged peroxisomal morphology, PEX19 protein was downregulated, consistent with lower gene expression (Fig. 1B, Supplementary Fig. 1C, D). In contrast, the peroxisome docking complex protein PEX14 was upregulated, despite unchanged gene expression (Fig. 1B, Supplementary Fig. 1C, D). Elevated levels of PEX14 in cardiac tissue was confirmed by immunofluorescence staining of cardiac tissue slices in young mice (Fig. 1C), which colocalized with the peroxisome lipid transporter ABCD3 in mouse heart sections (Supplementary Fig. 1E). Interestingly, elevated protein levels normalized in adult animals, coinciding with the development of cardiac dysfunction (Supplementary Fig. 1F) [7]. As a result of an altered peroxisomal protein import machinery, we observed elevated levels of the peroxisomal matrix protease LONP2 (Fig. 1D, Supplementary Fig. 1D). In contrast to the heart, PEX5 and PEX14 levels were not significantly altered in the liver, an organ that is particularly enriched in peroxisomes, indicating that peroxisomal changes are restricted to the organ in which mitochondrial function is also affected by the *Taz*-KD (Supplementary Fig. 1G) [23].

### Peroxisomal FAO is unchanged in *Taz*-KD heart

**Fig. 2:**
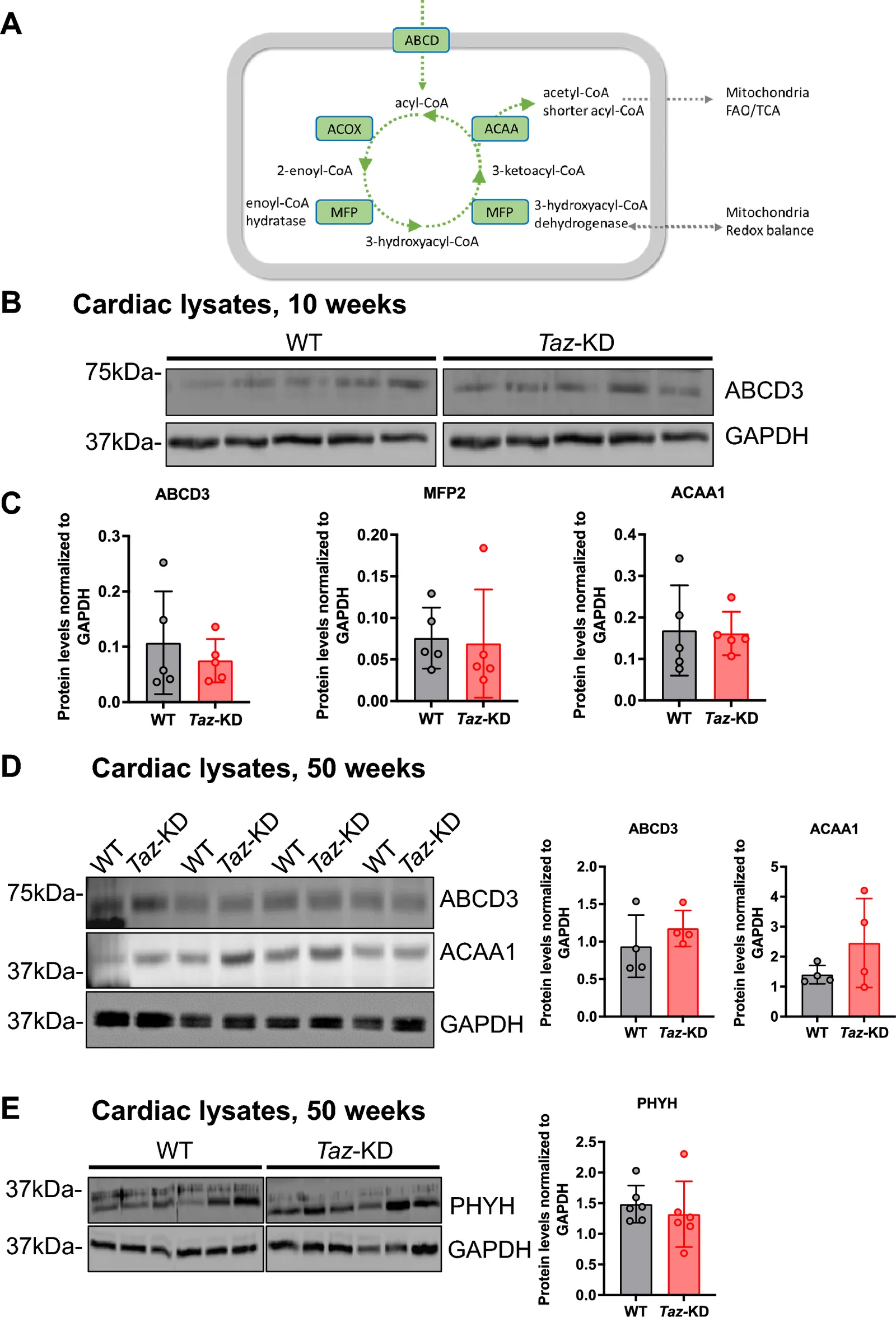
Peroxisomal FAO in the *Taz*-KD mouse heart. **A** Cartoon of peroxisomal FAO. **B** Protein levels of peroxisomal ABCD3, analyzed by western blot in 10-week-old *Taz*-KD hearts (n = 5). **C** Quantification of western blots, as in (**B**) for ABCD3, MFP2 and ACAA1 (values are presented as mean ± SD, n = 5). **D** Western blot analysis of peroxisomal FAO enzymes in 50-week-old *Taz*-KD hearts (n = 4). Quantifications are presented as mean ± SD, n = 4. **E** Western blot analysis of PHYH in 50-week-old *Taz*-KD hearts, with quantification (± SD, n = 6, right panel)

FAO is the most prevalent energy source of the healthy heart. *Taz*-deficiency substantially reduces mitochondrial fatty acid (FA) oxidation [61]. Peroxisomes possess a full enzymatic capacity for FAO and play a pivotal role in the α- and β-oxidation of long- and branched- chain fatty acids [62]. This raises the question whether the observed changes in peroxisomal protein import machinery serve to elevate the capacity for compensatory peroxisomal FA oxidation. FAs are imported into peroxisomes via ABCD transporters and subsequently oxidized. The product of the first oxidation (acyl-CoA oxidase, ACOX) is hydrated and undergoes a second oxidation, mediated by the multifunctional protein MFP2, and cleaved by the 3-ketoacyl-CoA thiolase ACAA1 (Fig. 2A). Gene expression of the transporter *Abcd1* was reduced at both ages, whereas *Abcd3* was upregulated in juvenile *Taz*-KD hearts and normalized in adult animals (Supplementary Fig. 2A). The gene expression of the oxidases *Acox1* and *Acox3* was reduced, whereas *Acox2* was elevated in both age groups. Other genes, including *Mfp2* and *Phyh,* remained unaltered in juvenile hearts, and only *Mfp2* decreased in adult animals (Supplementary Fig. 2A). Despite alterations in gene expression, protein levels of ABCD3 remained unaltered in 10-week and 50-week-old *Taz*-KD hearts (Fig. 2B-D). Also, immunofluorescence staining revealed no change in the number or intensity of ABCD3 positive particles in 10-week-old hearts (Supplementary Fig. 2B, C). The multifunctional protein MFP2 was unaltered in adult heart tissue, and ACAA1 remained unaltered in both age groups (Fig. 2C, D, Supplementary Fig. 2D). Finally, we also tested the peroxisome phytanoyl-CoA 2-hydroxylase PHYH, which is involved in the detoxification of phytanic acid via β-oxidation [101]. Also, this enzyme showed no genotype-specific difference in adult mouse hearts (Fig. 2E, Supplementary Fig. 2E). Together, these data indicate that despite alterations in gene expression in juvenile animals, protein levels of peroxisomal β-oxidation remain largely unchanged in *Taz*-KD hearts. Similar results were obtained in the liver, where ABCD3 and the 3-ketoacyl-CoA thiolase ACAA1 protein expression are unaltered, while PHYH expression is elevated (Supplementary Fig. 2F).

### Role of peroxisome catalase in preventing excess ROS

**Fig. 3:**
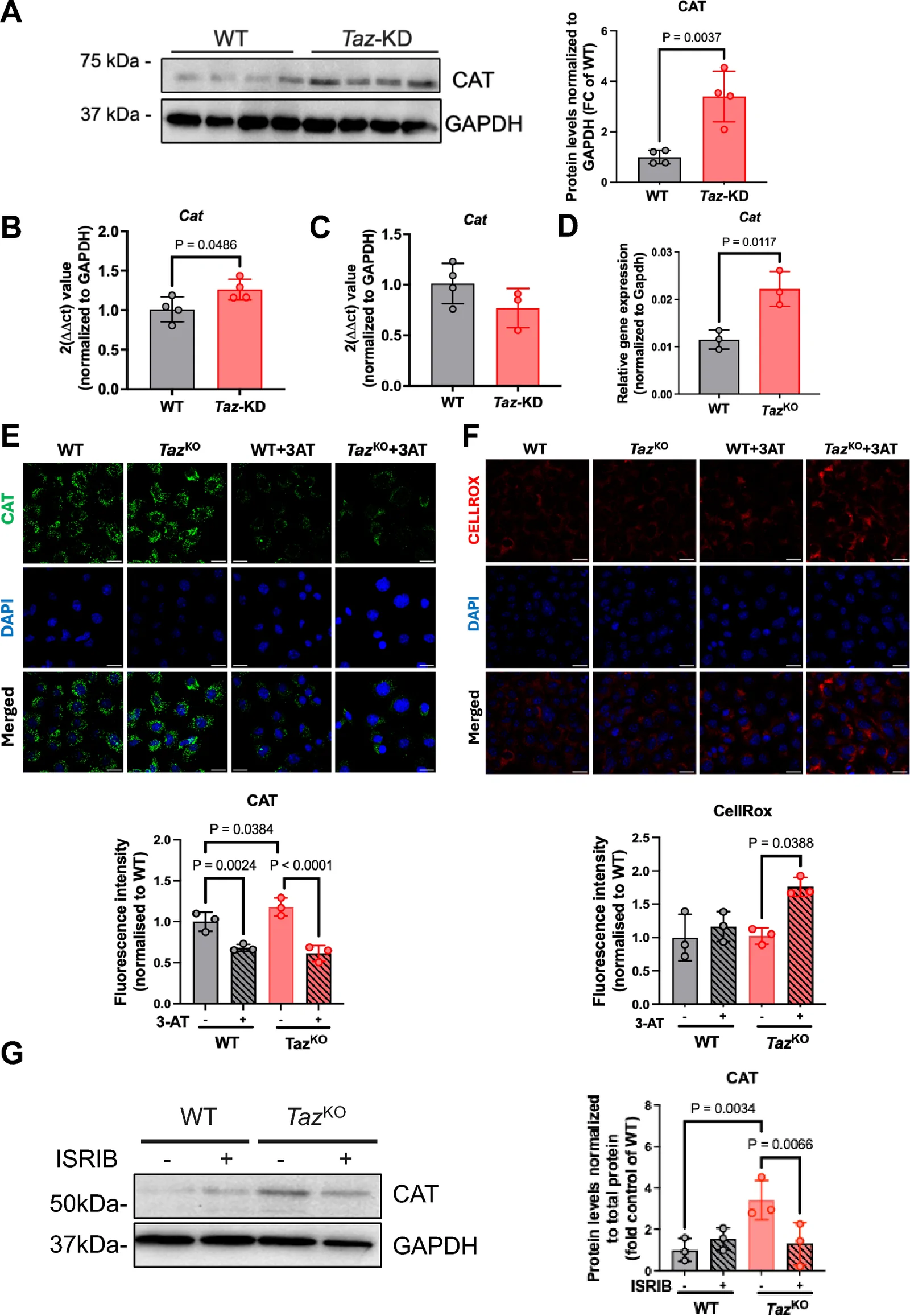
Peroxisomal catalase levels in *Taz*-KD mouse tissue. **A** Western blot analysis of catalase and GAPDH in 20-week-old heart tissue. Quantification of catalase, normalized to GAPDH in the right panel (values are presented as mean ± SD, n = 4). **B** qPCR analysis of catalase normalized to *Gapdh* in 10-week-old heart tissue. **C** qPCR analysis of cardiac catalase normalized to *Gapdh* in 50-week-old mice, n = 3. **D** Gene expression of catalase in *Taz*^KO^ MEF compared to WT (values are presented as mean ± SD, n = 3, statistical significance was determined with unpaired Student’s t-test). **E** Immunofluorescence of anti-catalase antibody (green) in MEF treated with 10 mM 3-AT or left untreated. Nuclei stained with DAPI (blue). Right panel: quantification of three experiments. Quantification in the right panel (values are presented as mean ± SD, n = 3 statistical significance was determined with two-way ANOVA followed by Tukey's multiple comparisons test). **F** Cell Rox fluorescence in MEF treated with 10 mM 3-AT or left untreated. Nuclei stained with DAPI (blue). Right panel: quantification of three experiments (values are presented as mean ± SD, n = 3, two-way ANOVA followed by Tukey's multiple comparisons test). **G** Western blot analysis of catalase and GAPDH as a control in *Taz*.^KO^ MEFs upon treatment with 450 µM ISRIB. Quantification of catalase, normalized to GAPDH in the right panel (values are presented as mean ± SD, n = 3)

Peroxisomal catalase converts H_2_O_2_ originating from FAO into H_2_O and O_2_. In the context of mitochondrial dysfunction, catalase plays an important role in maintaining cellular redox homeostasis [54, 65]. Western blot analysis revealed increased protein amounts in young *Taz*-KD hearts, which was normalized in aged *Taz*-KD mice (Fig. 3A, Supplementary Fig. 3A), in agreement with previous data from our laboratory [7]. These increased catalase protein levels were driven by elevated catalase gene expression in young mice, which normalized in aging animals (Fig. 3B, C). Also in kidney, adipose tissue and liver, catalase levels were modestly increased (Supplementary Fig. 3B-D). To gain further mechanistic insights, we employed mouse embryonic fibroblasts (MEFs) with deletion of the *Taz* gene (*Taz*^KO^), which largely recapitulates genetic and metabolic alterations of BTHS cardiac myocytes [61]. Also here, catalase mRNA (Fig. 3D) and protein levels were elevated (Fig. 3E). Aminotriazole (3-AT) is a specific catalase inhibitor that irreversibly binds to its catalytic site, causing aggregation and degradation of the inactivated protein [100]. In fact, 3-AT substantially decreased catalase levels in WT and *Taz*^KO^ MEF detected by immunofluorescence (Fig. 3E). The elimination of catalase protein was further confirmed by western blotting (Supplementary Fig. 3E) and resulted in a complete elimination of catalase activity (Supplementary Fig. 3F).

To assess the role of catalase on ROS elimination in WT and *Taz*^KO^ MEF, we employed CellROX, a whole-cell reporter of oxidative stress, which is rather unspecific for certain oxidant species. While in the absence of 3-AT, CellROX fluorescence was similar between the genotypes, a substantial increase in oxidative stress was observed in the presence of 3-AT in *Taz*^KO^, but not WT MEFs (Fig. 3F), indicating that catalase upregulation is required to prevent an increase in ROS in *Taz*^KO^ MEF, while in WT MEF, NADPH-coupled H_2_O_2_-eliminating systems are sufficient to prevent oxidative stress. We have shown before, that *Taz*-deficient mitochondria in *Taz*-KD mouse hearts and in *Taz*^KO^ MEF induce the activation of the ISR [61]. We therefore tested if the ISR pathway may contribute to the regulation of peroxisomal catalase. Treatment of *Taz*^KO^ MEFs with the ISR inhibitor ISRIB strongly prevented the elevation of catalase levels, indicating that peroxisomal remodeling, to some extent indeed depends on ISR signaling (Fig. 3G).

**Fig. 4:**
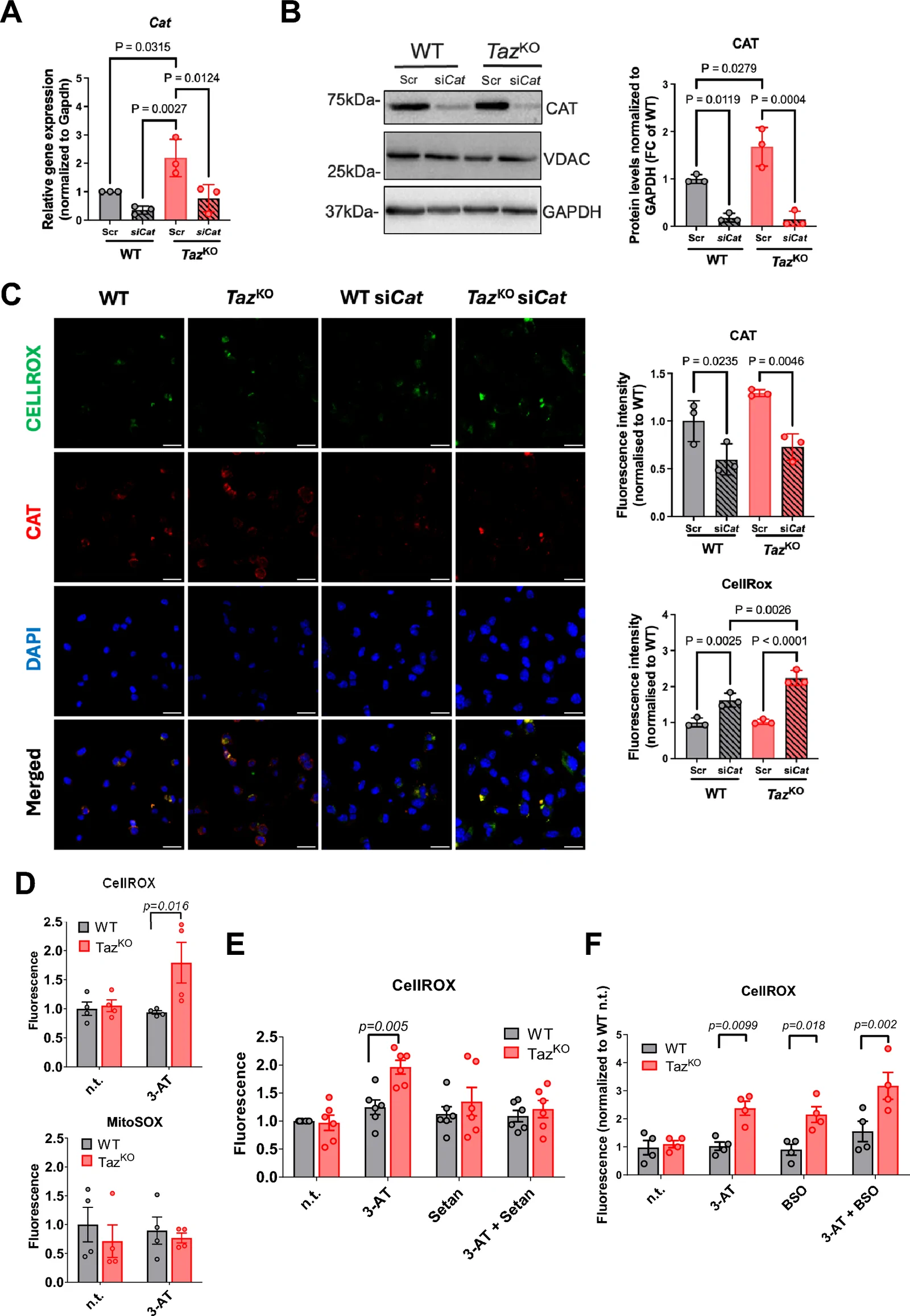
Catalase knockdown in *Taz*^KO^ MEFs. **A** qPCR analysis of catalase gene expression normalized to Gapdh in MEF transfected with siRNA against catalase or non-targeting control. Values are presented as mean ± SD, n = 3. Statistical significance was determined using two-way ANOVA followed by Dunnett’s multiple comparisons test. **B** Western blot analysis of catalase, VDAC, and GAPDH in MEF transfected with siRNA against catalase or non-targeting control. Values are presented as mean ± SD, n = 3, and statistical significance was determined with two-way ANOVA followed by Tukey's multiple comparisons test. **C** Cell ROX fluorescence (green) in MEF transfected with siRNA against catalase or non-targeting control. Catalase was labeled with anti-catalase antibody (red) and nuclei were stained with DAPI (blue, left panel). Quantification of catalase and CellROX fluorescence intensity in n = 3 experiments (lower panels). Values are presented as mean ± SD; statistical significance was determined with two-way ANOVA followed by Tukey's multiple comparisons test. **D** Analysis of cytosolic ROS via CellROX deep red and mitochondrial ROS via MitoSOX red in MEF upon treatment with 3-AT. Quantification of MitoSOX and CellROX fluorescence intensity in n = 4 experiments. Values are presented as mean ± SD; statistical significance was determined two-way ANOVA. **E** CellROX deep red fluorescence in MEF upon treatment with 10 mM 3-AT, 1 µM Setanaxib (Setan) or both. Quantification of fluorescence intensity in n = 6 experiments. Values are presented as mean ± SD; statistical significance was determined two-way ANOVA. **F** Quantification of CellROX deep red fluorescence in MEF upon treatment with 10 mM 3-AT, 150 µM BSO or both. Quantification of fluorescence intensity in n = 4 experiments. Values are presented as mean ± SD; statistical significance was determined with two-way ANOVA

To confirm our findings with 3-AT, we used siRNA-mediated knockdown of catalase gene expression, which also lowered catalase protein levels in WT and *Taz*^KO^ cells (Fig. 4A, B). Immunofluorescence confirmed lower catalase levels in si*Cat* treated samples (Fig. 4C). CellROX fluorescence was elevated specifically in *Taz*^KO^ cells, confirming results with 3-AT mediated catalase inhibition (Fig. 4C). Having established that peroxisomal catalase serves to protect from cytosolic ROS, we next wished to further delineate the source and type of ROS that catalase upregulation may protect from in tafazzin-deficiency. To this end, we employed the mitochondria-specific superoxide (^**.**^O_2_^−^) reporter MitoSOX in the same experiment as CellROX. At baseline, mitochondrial^**.**^O_2_^−^ was similar in *Taz*^KO^ compared to WT MEFs, similar to our findings in isolated cardiac mitochondria from *Taz*-KD vs. WT mice in our previous studies [7]. While oxidative stress in the cytosol increased upon catalase inhibition with 3-AT in *Taz*^KO^, but not WT MEFs (in agreement with our previous results in Fig. 3F), no increase of mitochondrial^**.**^O_2_^−^ was observed (Fig. 4D, Supplementary Fig. 4A). These data indicate that catalase upregulation is required to primarily prevent cytosolic ROS elevation in *Taz*^KO^ MEFs.

NADPH oxidases (NOX) are major sources of cytosolic ROS in the heart, and elevated NOX drive myocardial hypertrophy, fibrosis, and dysfunction in heart failure [60]. In addition, also monoamine oxidases MAO-A, MAO-B, or iNOS were reported to contribute to oxidative stress in heart failure [31, 49]. However, none of these genes showed increased expression in *Taz*^KO^ vs. WT MEFs (Supplementary Fig. 4B). We next addressed, whether inhibiting these sources of cytosolic ROS would relieve the ROS burden upon catalase inhibition. Interestingly, simultaneous inhibiting NOX1 and NOX4 using the specific inhibitor Setanaxib resulted in a normalization of ROS levels upon catalase inhibition with 3-AT (Fig. 4E, Supplementary Fig. 4C). These data indicate that catalase upregulation in *Taz*-deficient cells primarily protects from oxidative stress in the cytosol. Reducing the cytosolic ROS burden by inhibiting NADPH oxidases alleviates overall stress levels in *Taz*^KO^ MEFs, which needs to be compensated by upregulation of catalase.

Therefore, together with the ISR-mediated elevation of GSH biosynthesis, two compensatory mechanisms prevent ROS in *Taz*^KO^ MEFs. Both pathways combine to form an effective protection against oxidative damage in BTHS. We experimentally challenged ROS compensation in *Taz*^KO^ MEFs by using the catalase inhibitor 3-AT and an established inhibitor of GSH biosynthesis. Buthionine sulfoximine (BSO) is a potent, specific inhibitor of the glutamate-cysteine ligase (GCL) [36]. This enzyme catalyzes the rate-limiting step in the de novo biosynthesis of glutathione. Interestingly, catalase inhibition and inhibition of GSH-dependent antioxidative pathways induced ROS to a similar extend, indicating that both pathways equally contribute to ROS protection (Fig. 4F, Supplementary Fig. 4D).

### Catalase upregulation protects from oxidative stress in *Taz*-deficient cells

**Fig. 5:**
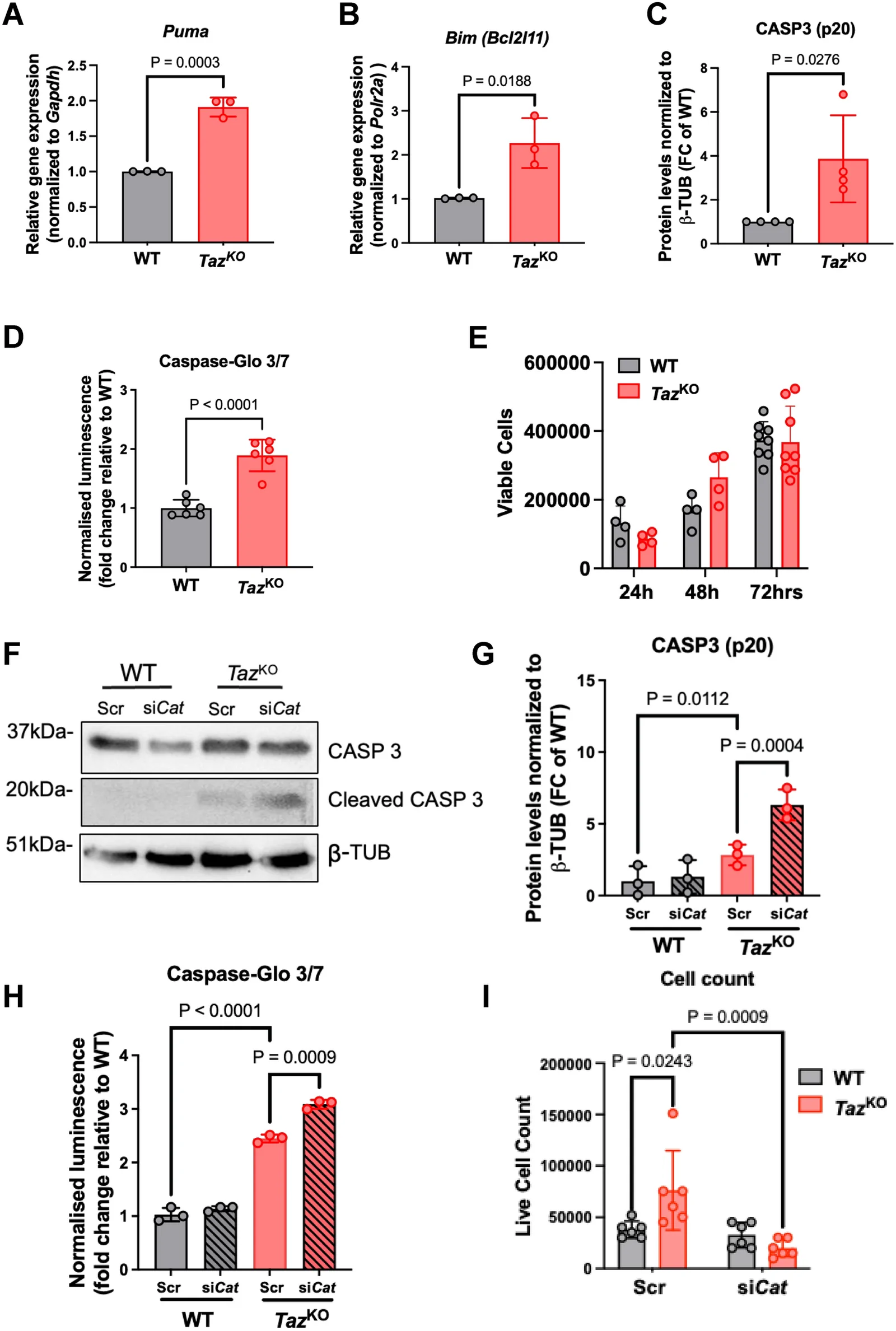
Role of peroxisomal catalase in *Taz*^KO^ MEFs. **A-B** qPCR analysis of *Puma* and *Bim (Bcl2l11)* mRNA expression levels were normalized to *Gapdh and Polr2a*. Values represent mean ± SD (n = 3). Statistical significance was assessed using an unpaired two tailed Student’s t-test. **C** Densitometric quantification of cleaved Caspase-3 protein levels in WT and *Taz*^KO^ values normalized to TUBULIN and are shown as mean ± SD (n = 3) Statistical significance was determined using unpaired two tailed Student’s t-test. **D** Analysis of caspase 3 cleavage activity measuring the conversion of luciferase coupled caspase substrate, liberating aminoluciferin, which is converted by luciferase generating a luminescent signal**.** (n = 6, mean ± SD, significance was determined by Student’s t-test). **E** Quantification of viable cells after growth of 30.000 cells for the indicated time points (n = 4–8 for individual time points, mean ± SEM). **F** Western blot analysis of the cleaved caspase p20 product, Caspase 3 and TUBULIN in MEFs transfected with siRNA against catalase or non-targeting control. **G** Quantification of three independent experiments, shown in Fig. 5F. Values are presented as mean ± SD, n = 3, statistical significance was determined with two-way ANOVA followed by Tukey's multiple comparisons test. **H** Analysis of caspase 3 activity as in Fig. 5D in WT and *Taz*^KO^, transfected with *siCat* or non-targeting RNA. Values are presented as mean ± SD, n = 3, statistical significance was determined with two-way ANOVA followed by Tukey's multiple comparisons test. **I** Total cell number of *Taz*^KO^ and control MEF, transfected with *siCat* or non-targeting RNA, treated with 20 µM H_2_O_2_ for 24 h. Values are presented as mean ± SD, n = 3, statistical significance was determined with two-way ANOVA followed by Tukey's multiple comparisons test

Excessive ROS can damage proteins, lipids and nucleic acids and eventually induce apoptotic cell death [86]. Apoptosis is a genetic program, culminating in the induction of proteins of the BCL2 protein family. Two genes of this family, *Puma (Bbc3)* and *Bim (Bcl2l11)* are slightly upregulated in *Taz*^KO^ MEF (Fig. 5A, B). Apoptosis is mediated by activation of a cascade of cysteine proteases, called caspases, which are expressed as procaspases and require proteolytic processing for activation [80, 89]. Interestingly, we detected a slight but significant elevation of the cleaved caspase 3 p20 fragment in *Taz*^KO^ MEF cell lysates by western blotting (Fig. 5C), in agreement with increased caspase 3 activity in a luciferase-based luminescence assay (Fig. 5D). The caspase 3 activity did not affect cell proliferation in *Taz*^KO^ MEF, indicating that excessive activation of apoptosis is prevented (Fig. 5E). Catalase knockdown increased the levels of active p20 caspase 3 fragment (Fig. 5F, G) and caspase 3 activity (Fig. 5H). Exogenous H_2_O_2_ led to a concentration-dependent increase in cell death in WT and *Taz*^KO^ MEFs, but *Taz*^KO^ MEFs tended to tolerate higher H_2_O_2_ concentrations. When exposing WT and *Taz*^KO^ MEFs to 20 µM H_2_O_2_, catalase knockdown increased the induction of cell death in *Taz*^KO^, but not WT MEFs (Fig. 5I). These data indicate that upregulation of catalase protects *Taz*^KO^ MEFs from cell death in response to endogenous, but also exogenous ROS.

### Role of plasmalogens in stress compensation, induced by mitochondrial dysfunction

**Fig. 6:**
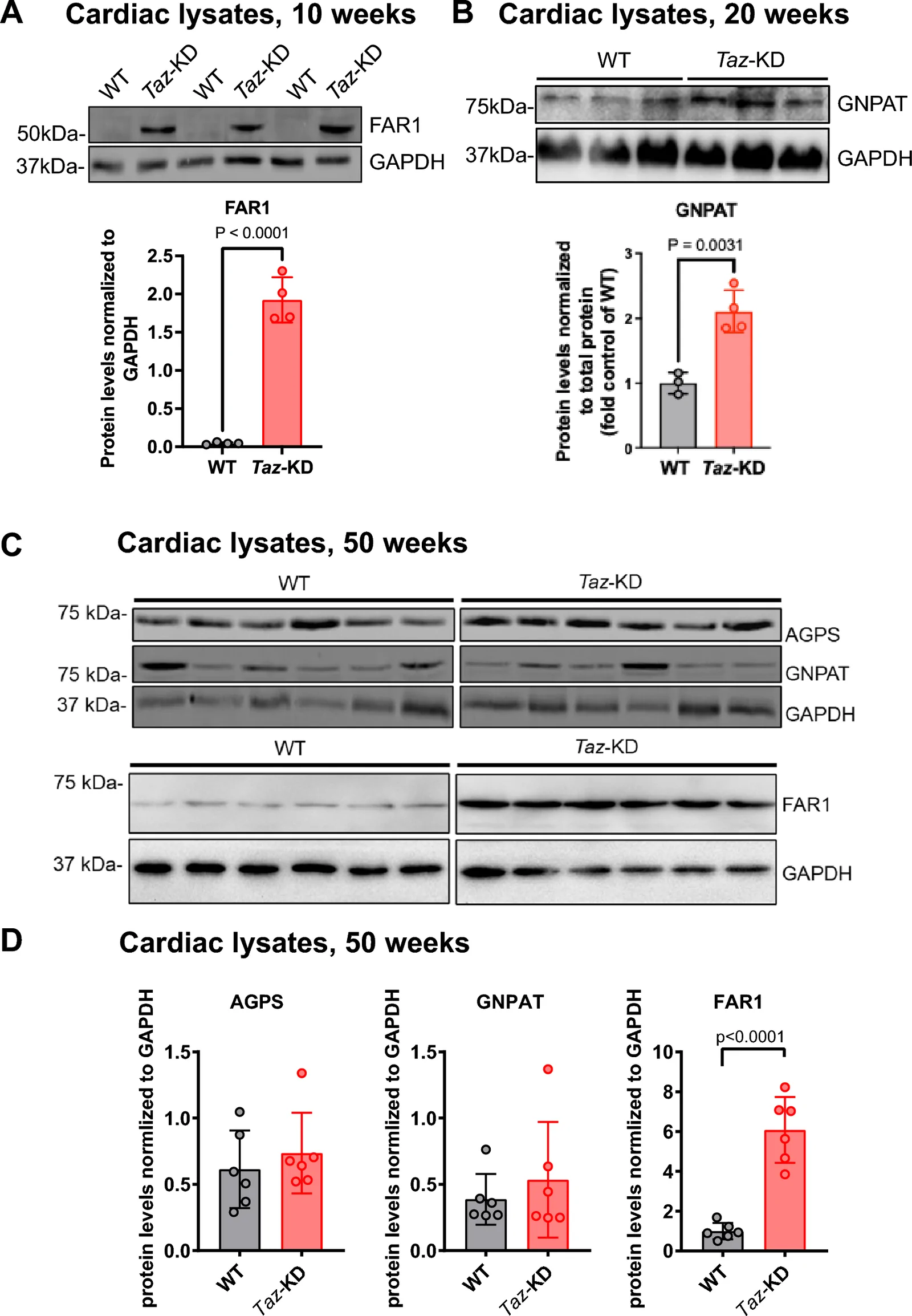
Plasmalogen biosynthesis in *Taz*-KD mouse heart. **A** Western blot of FAR1 compared to GAPDH In 10-week-old hearts and quantification via densitometric analysis. Values are presented as mean ± SD, n = 4, statistical significance was determined with Student’s t-test. **B** Western blot and quantification of GNPAT 20-week-old hearts. Values are presented as mean ± SD, n = 4. **C** Western blot of indicated proteins compared to GAPDH in 50-week-old hearts. **D** Quantification of western blots in (**C**). Values are presented as mean ± SD, n = 6

Another important function of peroxisomes is plasmalogen biosynthesis. Plasmalogens play an important role for the organization and stability of cell membranes [44, 81]. We observed substantial alterations in the acyl chain composition of ether-phosphatidylcholine and phosphatidylcholine based plasmalogens in cardiac tissue of the *Taz*-KD mouse [38]. We therefore analyzed the three peroxisomal enzymes GNPAT, AGPS and FAR1, which are involved in the peroxisomal arm of plasmalogen biosynthesis. In hearts of young mice, protein levels of the rate limiting enzyme FAR1 were markedly elevated, as described before [55] (Fig. 6A). We also noticed a substantial upregulation of GNPAT in young animals (Fig. 6B). In aged hearts, FAR1 protein expression is still upregulated, whereas GNPAT and AGPS were unaltered (Fig. 6C, D). Elevated levels of FAR1 were not driven by elevated gene expression (Supplementary Fig. 5A). This is consistent with previous data showing that FAR1 is mostly regulated on protein level [43]. FAR1 is also independent of catalase levels as knockdown of catalase does not affect FAR1 levels in *Taz*^KO^ MEFs (Supplementary Fig. 5B, 5C). Gene expression of *Agps1* was slightly downregulated, whereas *Gnpat* gene expression remained unchanged (Supplementary Fig. 5A). Elevated FAR1 protein levels were recapitulated in the liver of 50 weeks-old *Taz*-KD mice, where AGPS protein expression was also slightly upregulated (Supplementary Fig. 5D).

**Fig. 7:**
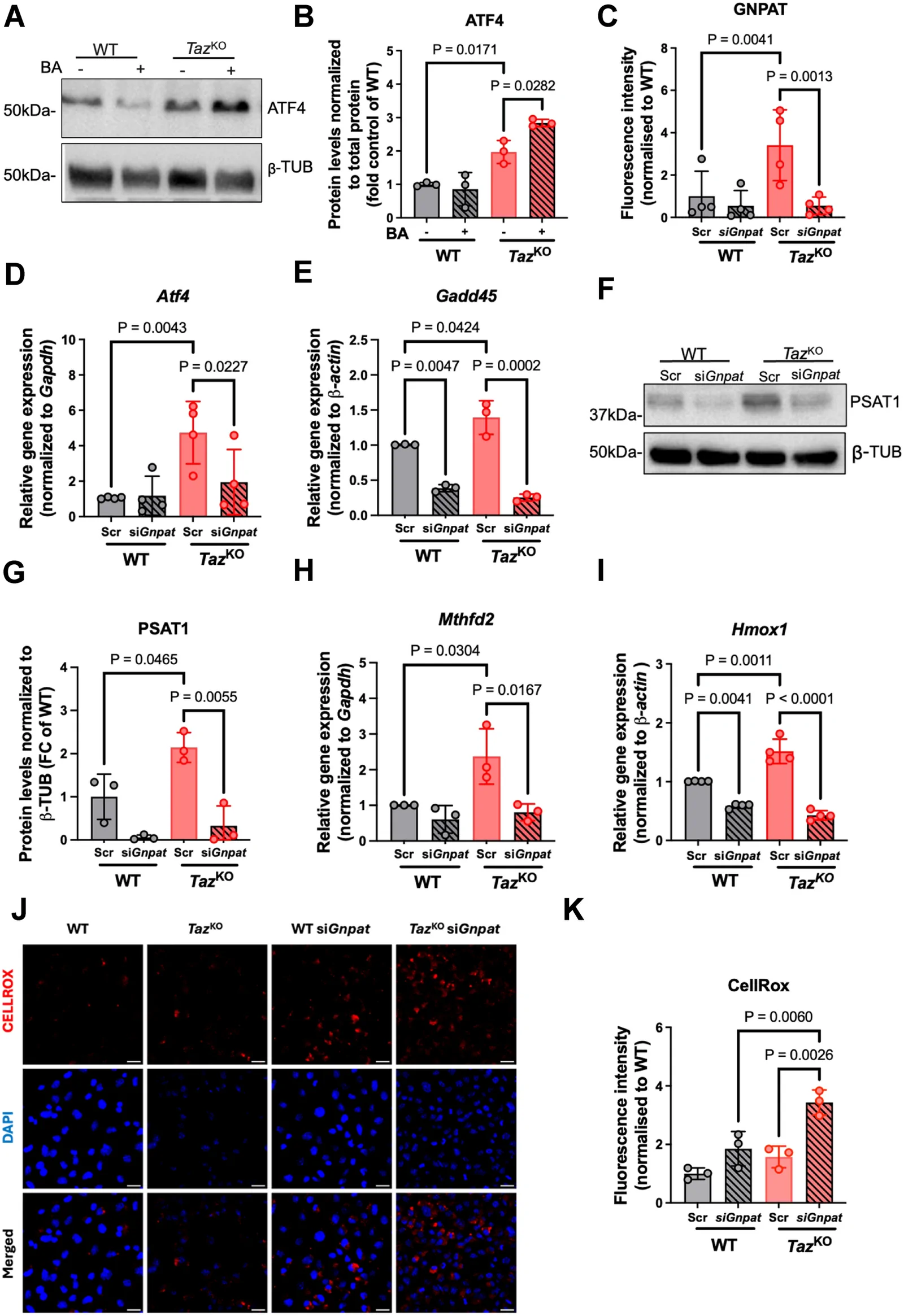
Role of plasmalogen biosynthesis in *Taz*^KO^ MEFs. **A** MEF were treated with 20 µM batylalcohol for 72 h and subjected for western blot analysis for ATF4 and TUBULIN as a control. **B** Quantification of experiments as described in (A). Values are presented as mean ± SD, n = 3. **C** Quantification of immunofluorescence analysis of GNPAT in MEF, transfected with siRNA against *Gnpat* or with non-targeting control. Values are presented as mean ± SD, n = 4, statistical significance was determined two-way ANOVA followed by Tukey's multiple comparisons test. **D**, **E** qPCR analysis of indicated gene expression in MEF, transfected with siRNA against GNPAT or with non-targeting control. Values are presented as mean ± SD, n = 3–4, statistical significance was determined two-way ANOVA followed by Tukey's multiple comparisons test. **F** Western blot analysis of PSAT in MEF, transfected with siRNA against GNPAT or with non-targeting control. **G** Quantification of experiment as described in (**F**). Values are presented as mean ± SD in right panel, n = 3, statistical significance was determined two-way ANOVA followed by Tukey's multiple comparisons test. **H** *Mtfhd2* and **I** *Hmox1* gene expression after si*Gnpa*t knockdown. Values are presented as mean ± SD, n = 5, statistical significance was determined two-way ANOVA followed by Tukey's multiple comparisons test. **(J)** CellROX fluorescence (red) in MEF transfected with siRNA against GNPAT or non-targeting control. Nuclei were stained with DAPI (blue, left panel). **K** Quantification of CellROX fluorescence intensity in n = 3 experiments (lower panels). Values are presented as mean ± SD; statistical significance was determined two-way ANOVA followed by Tukey's multiple comparisons test

The final steps of plasmalogen biosynthesis takes place in the ER. Here, plasmalogens reside in the same compartment as the ER resident signaling kinase PERK, which is responsible for the activation of the ISR stress response in BTHS [61]. We therefore interrogated a possible interconnection between plasmalogens and ISR signaling in BTHS. Administration of batylalcohol, a precursor of plasmalogen biosynthesis, is an established method to elevate plasmalogen levels [99]. We found that batylalcohol administration strongly induced ATF4, particularly in *Taz*-deficient cells with dysfunctional mitochondria (Fig. 7A, B). These data pointed towards a supportive role of plasmalogens for the activation of the ISR pathway in BTHS [61].

To support this concept, we investigated the effect of reduced plasmalogen biosynthesis on ISR signaling in *Taz*-deficient MEF. In *Taz*^KO^ MEFs, besides elevated FAR1, also GNPAT was significantly elevated as detected by immunofluorescence and western blotting (Fig. 7C, Supplementary Fig. 6A). Elevated GNPAT protein was a result of increased gene expression (Supplementary Fig. 6B). As FAR1 protein levels are regulated independently of gene expression, we targeted *Gnpat* via siRNA [43]. qPCR confirmed downregulation of *Gnpat* mRNA to a similar degree in *Taz*^KO^ and control MEFs (Supplementary Fig. 6B), which also led to downregulation of GNPAT protein levels (Fig. 7C). We found that interfering with peroxisomal plasmalogen biosynthesis through si*Gnpat* knockdown blunted activation of the ISR, since *Atf4* gene expression was normalized in si*Gnpat* knockdown cells (Fig. 7D) [61]. To substantiate these findings, we also tested gene expression of the ATF4-dependent transcription factors *Gadd45* and *Chop1.* Both genes are significantly upregulated in *Taz*^KO^ MEFs, similar to our previous findings [61]. Notably, si*Gnpat* decreased GADD45 and CHOP1 signaling in *Taz*^KO^ and control MEFs, indicating a strong reduction of ISR signaling (Fig. 7E, Supplementary Fig. 6C). These data indicate that increased peroxisomal plasmalogen biosynthesis supports the ISR.

We previously revealed that the ISR and ATF4 governed metabolic rewiring of enzymes that divert glucose from glycolysis into the biosynthesis of serine [61]. Accordingly, si*Gnpat* also blunted the upregulation of phosphoserine aminotransferase (PSAT), the hallmark enzyme of the serine biosynthesis pathway on mRNA (Supplementary Fig. 6D) and protein (Fig. 7F, G) level. The serine pathway fuels the 1C metabolism, which supplies multiple metabolic pathways, including nucleotide metabolism and GSH biosynthesis. Gene expression of the hallmark enzyme MTHFD2 is strongly increased in *Taz*-KD hearts and *Taz*^KO^ MEF, as shown before (Fig. 7H) [61]. Following reduced ATF4 signaling, si*Gnpat* also strongly blunted *Mthfd2* elevation in *Taz*^KO^ MEFs (Fig. 7H). As the elevated serine pathway and 1C metabolism in BTHS served to elevate GSH production [61], these data indicate that plasmalogen biosynthesis supports redox defense mechanisms. In line with these findings, elevated levels of heme oxygenase (*Hmox1*) and NAD(P)H:quinone oxidoreductase 1 (*Nqo1*), which are both involved in ROS compensation and joint targets of ATF4 and the transcription factor NRF2 are reduced upon si*Gnpat* (Fig. 7I, Supplementary Fig. 6E) [32, 59]. These data indicate that by supporting ISR activation, plasmalogen biosynthesis contributes to ROS defense. In this context, we also analyzed if plasmalogen also directly regulated catalase gene expression. However, plasmalogen administration was not sufficient to induce catalase gene or protein expression in MEFs (Supplementary Fig. S6F, S6G). We next tested if si*Gnpat* affects redox protection. Knockdown of *Gnpat* increased cytosolic ROS, measured by CellROX (Fig. 7J, K). These results highlight a novel role of peroxisome’s protective role in mitochondrial dysfunction by supporting ROS defense mechanisms.

## Discussion

**Fig. 8:**
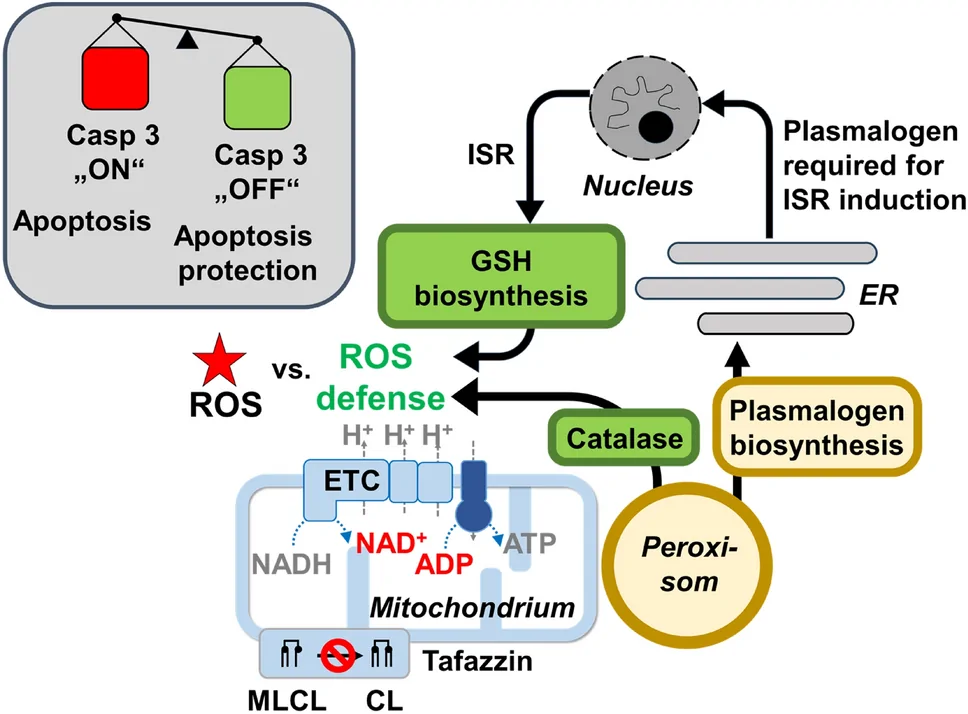
Peroxisomal catalase and plasmalogen synthesis converge to boost the anti-oxidative capacity in *Taz*-deficient cells

In BTHS cardiomyopathy, defective CL remodeling affects the respiratory chain, the Krebs cycle and oxidation of fatty acids, which is the most relevant cardiac fuel [1, 7, 26, 27, 33, 67, 93]. Defects in mitochondrial Ca^2+^ signaling causes oxidation of the NAD(P)H redox buffers and relates to contractile dysfunction and arrhythmias in iPSC-CM and murine models of BTHS [7, 103, 105]. Mitochondrial dysfunction is compensated by a metabolic switch towards glucose and glutamate uptake and utilization mediated by the ISR [12, 14, 61, 63]. The ISR compensates not only deficient ATP production but also boosts the anti-oxidative capacity. Peroxisomes are known to physically associate with mitochondria to allow for the exchange of metabolic intermediates and harbor relevant amounts of catalase to ameliorate oxidative stress [29, 56, 57, 75, 90]. However, their alterations in mitochondrial diseases and in particular, in BTHS cardiomyopathy were previously unknown. Here, we identify remodeling of the peroxisomal proteome of a murine BTHS model (*Taz*-KD mice) with a moderate cardiomyopathy that closely resembles BTHS patients [7, 15]. While peroxisomal morphology and fatty acid metabolism are largely unchanged, upregulation of catalase and enzymes involved in plasmalogen synthesis converge to boost the anti-oxidative capacity in *Taz*-deficient cells (Fig. 8). Of particular note, we identified that increased expression of GNPAT, a key enzyme in plasmalogen biosynthesis, was required for inducing the ISR with its central transcription factor ATF4, which is known to protect from heart failure development through bolstering antioxidative capacity [106]. Together, our data provide novel insights into the mitochondrial-peroxisomal interplay in BTHS, which may be leveraged for novel treatment approaches for this specific disease, but potentially also other mitochondrial cardiomyopathies.

The heart is one of the body's most energy-intensive organs, and both inherited and acquired cardiovascular diseases are often associated with disruptions in energy metabolism [3]. Cardiac metabolism depends on a continuous supply of oxygen and nutrients, along with the efficient removal of metabolic by-products through the coronary circulation. Adequate nutrient availability and waste clearance are essential for proper mitochondrial energy production. Consequently, disturbances in nutrient delivery and waste disposal such as in coronary artery disease (CAD) are considered major contributors to heart failure [40, 68]. Moreover, the relationship between coronary blood flow, mitochondrial dysfunction, and heart failure is complex and interdependent. Interestingly, CAD is frequently associated with mitochondrial dysfunction. Respiratory chain activity is reduced causing reduced energy conversion and inducing a metabolic shift to elevated glycolytic rates in CAD tissue [2]. While mitochondrial dysfunction impairs cardiac function, cardiac disease can further influence coronary blood flow through neurohumoral mechanisms [40, 68]. The interplay between mitochondrial dysfunction, coronary function, and nutrient availability in inherited mitochondrial diseases such as BTHS is not yet well characterized and constitutes a promising avenue for future studies.

### Role of peroxisomes for cardiac function

Peroxisomes play an essential role in cardiac function. Refsum's disease and Rhizomelic Chondrodysplasia Punctata (RCPD) are peroxisomal disorders, often caused by a mutation in the import receptor PEX7 or its most important substrate GNPAT. Patients commonly present with fatal heart failure, implying an important role of peroxisomes in maintaining heart function [102]. Arrhythmias and QRS complex prolongation were observed in *Gnpat* knockout mice, which was normalized by oral supplementation of plasmalogens [99]. In Zellweger syndrome, caused by a defect in the peroxisomal import receptor PEX5, a severe, multi-organ dysfunction with a predominance of neuro-sensory symptoms limits survival before the onset of a cardiac phenotype. In a parallel study, we observed that cardiomyocyte-specific PEX5 deletion causes dilated cardiomyopathy and heart failure in mice, supporting a critical role of peroxisomes for heart function [42]. A role of PEX5 in cardiac hypertrophy was also demonstrated in a rat model of pressure overload, where PEX5 protein levels declined over time. PEX5 overexpression alleviated cardiomyocyte hypertrophy, and reducing PEX5 levels exacerbated hypertrophy [104]. These data demonstrate a pivotal role for peroxisomes in heart function and inspire us to examine their possible role in compensating for mitochondrial dysfunction in BTHS.

### Peroxisome protein remodeling in BTHS cardiomyopathy

In the current study, we show that mitochondrial dysfunction induced by *Taz*-deficiency induces adaptations in the peroxisomal protein content. This aligns with our previous observation that peroxisomal protein content varies in different mouse organs and dynamically adapts to specific metabolic requirements [16]. Our analyses revealed a substantial increase in the protein PEX14 in young animals, which plays a pivotal role in facilitating the import of proteins into nascent peroxisomes. As a result of the dynamic adaptation of the peroxisomal proteome, levels of catalase and LONP2 were elevated in *Taz*-KD mouse hearts.

LONP2 is a protease involved in the degradation of oxidatively damaged proteins, consistent with a role in stress defense [53]. LONP2 also regulates the peroxisomal proteome by removing metabolic enzymes, thus maintaining enzyme levels at an optimal level [5, 82]. LONP2 may also act as a chaperone during protein import, preventing the misfolding of imported proteins. Together, with alterations in the import machinery, these data indicate a specific alteration of peroxisomal proteome in *Taz*-KD hearts. Interestingly, these rather adaptive changes revert in aged animals, coinciding with the onset of cardiac dysfunction [7].

Peroxisomal FAO plays an essential role in the metabolism of dietary branched-chain fatty acids, which accumulate and induce cardiotoxic effects [37]. We therefore addressed whether the content of FAO enzymes is altered to compensate for the defect of mitochondrial FA oxidation. However, we did not find any evidence of a compensatory increase of peroxisomal FAO proteins (ABCD3, MFP2, ACAA1 or PHYH).

### Role of catalase upregulation in cardiac peroxisomes

Despite similar levels of FAO enzymes, catalase was upregulated in young *Taz*-KD mouse hearts. Elevated catalase levels were restricted to young *Taz*-KD animals, and a decrease in catalase coincided with the deterioration of heart function in this mouse model [7]. Our analyses with *Taz*^KO^ MEF showed a protective function of peroxisomal catalase. CL deficiency primarily affects mitochondrial function. Dysfunctional mitochondria can affect cytosolic ROS levels in several ways: Superoxide (^**.**^O_2_^−^) produced in the intermembrane space (in particular, by complex III) can diffuse directly into the cytosol, or^**.**^O_2_^−^ produced inside the matrix (by complexes I and III) is rapidly converted to hydrogen peroxide (H_2_O_2_) by the manganese-dependent superoxide dismutase (Mn-SOD). While^**.**^O_2_^−^ is usually contained within the matrix (unless released by opening of pores), H_2_O_2_ can freely diffuse across the mitochondrial membranes into the cytosol. In cardiac myocytes, mitochondria are effective whole-cell H_2_O_2_-scavenging organelles that substantially contribute to ROS scavenging also in the cytosol. Mitochondrial dysfunction can also affect cytosolic ROS scavenging when mitochondrial redox buffers are depleted by hampered supply of reducing equivalents, such as NADPH [24].

Our previous studies revealed oxidation of NAD(P)H in response to β-adrenergic stimulation in cardiac myocytes of young (10 week old) Taz-KD mice, and oxidation of NAD(P)H already at baseline in 20 or 50 week-old *Taz*-KD mice [7]. The underlying mechanism is a loss of the mitochondrial Ca^2+^ uniporter causing defective mitochondrial Ca^2+^ uptake, which results in a deficient Ca^2+^-mediated activation of Krebs cycle dehydrogenases that regenerate the redox state of NADH and NADPH. Since NADPH flux is key to fuel H_2_O_2_-eliminating enzymes in mitochondria, such as glutathione peroxidase or the thioredoxin/peroxiredoxin system, lack of NAD(P)H provision endangers redox compensation, particular under conditions of elevated cardiac energy demand [24]. Interestingly, free mitochondrial hydrogen peroxide (H_2_O_2_) levels, determined by the H_2_O_2_-sensitive genetic sensor mito-roGFP-Orp1, were not elevated in Taz-KD vs. WT mouse hearts [7].

As mitochondrial ROS in *Taz*^KO^ MEFs was never measured before, it was important to address mitochondrial ROS in comparison with cytosolic ROS. We therefore used two ROS sensitive dyes, i.e., MitoSOX for mitochondrial superoxide (^**.**^O_2_^−^) and the broad-spectrum ROS-indicator CellROX for the cytosol to determine the compartmentalization of ROS in the same experiment. No change in ROS burden was found in cytosol and mitochondria in the *Taz*^KO^ MEFs. Notably, in contrast to elevated levels of cytosolic ROS, catalase inhibition via 3-AT did not elevate mitochondrial superoxide levels in *Taz*^KO^ MEFs. This experiment indicates, that although the causative defect resides in the mitochondria, catalase is required to protect from elevated ROS in the cytosol. Experiments with the mitochondrial targeted genetic hydrogen peroxide sensitive sensor HyPer7 also indicated no elevated hydrogen peroxide levels in mitochondria (data not shown). Cytosol restricted oxidative stress favours the idea that failing redox compensation due to limited supply of mitochondrial reducing equivalents endanger the cytosolic redox compensation, requiring elevated catalase levels [24, 58]. We further tested the concept that exhaustion of ROS defense due to mitochondrial dysfunction may require elevated catalase to compensate cytosolic ROS. In particular NADPH oxidases (NOX) play a role in the pathophysiology of ventricular hypertrophy, the accompanying interstitial fibrosis and subsequent heart failure [11]. To test a role of NOX in the exhaustion of ROS defense in *Taz* deficient cells we used inhibitors against NOX, in the presence of catalase inhibitors. Interestingly, in presence of the NOX1 and NOX4 specific NOX inhibitor Setanaxib, we found, that catalase inhibition using 3-AT did not result in elevated ROS levels. These data indicate that NADPH oxidases may contribute to an elevated ROS burden, which needs to be rescued by the expression of catalase.

In this context, it may be relevant that ROS from mitochondria and NADPH oxidases can reinforce each other. For instance, it has been shown that mitochondrial ROS can increase NOX-dependent ROS formation, and also vice versa, ROS from NOX can trigger mitochondrial ROS [22, 73, 108]. Together with the observation that mitochondria are important to detoxify also cytosolic ROS in cardiac myocytes, it is not trivial to definitely delineate whether the initial increase in ROS starts in mitochondria, NOX or both [24]. However, we can conclude from our data that the upregulation of catalase protects the cell from oxidative stress in Barth syndrome.

Our findings in BTHS are in line with the observation of elevated peroxisomal catalase gene expression, protein levels and enzymatic activity in HCM and DCM patients [25]. Catalase overexpression ameliorates cardiac remodeling and dysfunction in a mouse model for angiotensin signaling induced dilated cardiomyopathy [85]. Catalase overexpression also improved cardiac function upon myocardial infarction [83]. In Langendorff perfused rabbit hearts, H_2_O_2_-mediated decrease in sarcomere shortening was alleviated by PPARγ agonists, which elevated catalase protein expression, and the cardioprotective effects were abolished by inhibition of catalase [13]. In the context of lipopolysaccharide induced myocardial damage, overexpression of catalase alleviated cardiac dysfunction and fibrosis by reducing apoptosis [84]. Finally, global overexpression of catalase targeted to mitochondria expands lifespan [91] and ameliorate cardiac dysfunction in response to aging [18, 21], pressure overload [19] and angiotensin infusion [20].

Considering the critical role of oxidative stress for the induction of cell death, we determined levels of cleaved caspase 3 in WT and *Taz*^KO^ MEF as an index for apoptotic cell death. In fact, we observed elevated levels of cleaved caspase 3 in *Taz*^KO^ MEF, which is in contrast to previous findings showing reduced apoptosis levels in immortalized lymphoblasts from BTHS patients [35]. As growth rates were similar in *Taz*^KO^ MEFs compared to control, we speculate that low grade caspase 3 cleavage does not yet reach the threshold for the full execution of apoptosis and cell death. We followed up on the concept of catalase affecting ROS induced apoptosis. Indeed, catalase inhibition revealed a protective function in *Taz*-deficient, but not WT cells, in line with the concept of peroxisomes playing an important role in protecting from apoptosis in BTHS [96, 107].

### Role of plasmalogens in BTHS

Plasmalogen biosynthesis is essential in the heart and plasmalogen levels are altered in heart failure [9]. Plasmalogens were among the most reduced lipid species in a patient cohort with acute myocardial infarction [95]. Dietary supplementation of plasmalogen precursors successfully reestablished plasmalogen levels and alleviated reperfusion injury in a rat myocardial infarction model [76]. However, whether plasmalogens help to maintain the redox homeostasis in BTHS cardiomyopathy is currently unclear. We and others have reported alterations in the plasmalogen content in the hearts of *Taz*-KD mice. While NMR data revealed reductions in the overall levels of plasmenylcholine, our data, using mass-spectrometry rather indicated changes in the plasmalogen acyl-chain composition [38, 55]. Importantly, the enzymes of plasmalogen biosynthesis were upregulated in *Taz*-KD mouse heart and liver and in *Taz*^KO^ MEF. However, the role of plasmalogens in controlling ROS and ROS-induced damage is controversial. Multiple studies showed a ROS-protective role of plasmalogens [97, 98, 111]. The vinyl-ether bond in plasmalogens is responsible for scavenging peroxy-radicals and oxidized poly unsaturated fatty acid (PUFA) products. Cells with higher plasmalogen levels are more resistant to H_2_O_2_, whereas protective actions are absent in cells lacking plasmalogens [110]. On the other hand, other studies contradict the simplistic ROS scavenging role of plasmalogens. Plasmalogen-derived oxidation products, especially 2-hydroxy-fatty aldehydes and 2-halo-fatty aldehydes, are reactive molecules that can exacerbate ROS damage by modifying amino groups of lipids and proteins [70, 94, 109]. Here, we found a novel mechanism how plasmalogens compensate an elevated ROS burden that extends beyond a direct involvement in ROS scavenging. *Gnpat* knockdown strongly blunted the ISR, indicating that plasmalogen biosynthesis is required to support ISR signaling. ISR signaling, in particular through the upregulation of the transcription factor ATF4, induces metabolic rewiring, including the biosynthesis of the protective ROS buffer GSH and induction of ROS scavenging enzymes, such as Nqo1 and Hmox1 [61]. Interestingly, we found that also catalase upregulation in *Taz*^KO^ MEF is dependent on ATF4 signaling. Whether this regulation is direct or indirect remains currently unexplored. Also, in acquired forms of heart failure induced by pressure overload, ATF4 is upregulated and ameliorates oxidative stress and cardiac dysfunction by upregulating enzymes that generate NADPH [106].

The mechanistic link how dysfunctional mitochondria induce ISR signaling and how plasmalogen biosynthesis supports ISR activation remains unclear. As the structure of plasmalogens as ether lipids makes this lipid species particularly vulnerable to oxidative stress, upregulated enzymes of plasmalogen biosynthesis might contribute to the high levels of oxidized phospholipids in *Taz*-deficient cell lines, lymphoblasts, mouse cardiac tissue and BTHS patients [48]. Oxidative stress is a key driver of the ISR [17, 61]. It can be speculated that elevated levels of oxidized plasmalogens are a key driver of ISR activation in *Taz*-deficient cells. In support for this hypothesis, we found that administration of ferrostatin, which specifically inhibits lipid peroxidation, strongly reduces ISR activation and the elevation of protein levels of ATF4, PSAT1 and MTHFD2 (data not shown) [77]. Plasmalogens may also play a more structural role in ISR activation, as plasmalogens and ISR activating kinases are both integral constituents of the ER membrane. In this context, it may be relevant that plasmalogens have a higher propensity to form nonlamellar structures [72]. It can be speculated that this property induces structural tension in the ER membrane and the membrane integrated ISR sensor kinases. Membrane tension may affect the threshold for dysfunctional mitochondria to induce ISR signaling. In conclusion, our data indicate that plasmalogens are required to maintain ISR signaling in the context of dysfunctional mitochondria in *Taz*-deficient cells. The ability of plasmalogens to affect stress response signaling might explain the controversial data in the literature whether plasmalogens rather eliminate ROS or further induce ROS generation [30, 46, 97, 98, 111].

We have previously shown that *Taz* deficiency induces stress response pathways in iPSC-CM, MEFs, mouse heart and skeletal muscle and in BTHS patient cardiac biopsies [61]. The activation of ISR is linked to a substantial activation of metabolic pathways (including serine pathway and 1C metabolism), which fuel the biosynthesis of GSH. Besides its role in controlling GSH biosynthesis and GSH-dependent peroxidases, our data also indicate a role of ISR in regulating the GSH-independent enzyme catalase in context of dysfunctional mitochondria. Both pathways combine to form an effective protection against oxidative damage in BTHS. We experimentally challenged ROS compensation in *Taz*^KO^ MEFs by using the catalase inhibitor 3-AT and the established inhibitor of GSH biosynthesis BSO. Catalase inhibition and inhibition of GSH-dependent antioxidative pathways induced ROS to a similar extend, indicating that both pathways equally contribute to ROS protection.

## Conclusions

Mitochondrial defects in BTHS, but also other mitochondrial cardiomyopathies induce heart failure primarily through energetic deficit and oxidative stress [74]. Cumulative evidence from preclinical and clinical studies suggest that the degree of oxidative stress may be a critical driver of disease progression in BTHS cardiomyopathy. Our data suggest that adaptive processes in peroxisomes, such as upregulation of catalase and enzymes involved in plasmalogen synthesis boost the antioxidative capacity in *Taz*-deficient cells. As an unexpected and novel finding, upregulation of plasmalogen synthesis is required to induce the ISR, including ATF4, which governs metabolic rewiring to sustain the provision of reducing equivalents required for anti-oxidative enzymes, as previously shown in BTHS [61] and other etiologies of heart failure [41, 106]. Together, these data provide unprecedented insights into the tight interplay between mitochondria and peroxisomes in a prototype mitochondrial disease that may help to design novel therapies to better treat these orphaned diseases [64].

## Materials and methods

### Mice

Animal experiments were performed according to the guidelines from the German Animal Welfare Act and approved by the Bayerisches Landesamt für Gesundheit und Lebensmittelsicherheit, Germany (AZ: 55.2.2–2535-804). To induce the expression of the shRNA construct, targeting the *Taz* gene, Doxycycline (625 mg/kg) was administered as part of the standard rodent chow to WT C57BL/6N mice and transgenic (ROSA26H1/tetO-shRNA:TAZ) animals.Doxycycline was withdrawn from female mice for 1 week before mating and during the mating period to avoid male infertility. Doxycycline treatment was resumed upon successful mating (copulatory plugs) and continued until the end of experiment.

### Cell culture and siRNA-mediated knockdown

Mouse embryonic fibroblasts (MEFs) were cultured in high-glucose DMEM (Gibco) supplemented with 10% fetal calf serum (Sigma), 1% penicillin/streptomycin (Gibco), and 4 mM glutamine (Gibco). They were transferred to a fresh 75 cm^2^ cell culture flask and maintained in a humidified incubator at 37 °C with 5% CO₂. Cells were passaged every two days once they reached 80–100% confluence. For passaging, the medium was discarded, and the flask was washed with 1 × PBS. Cells were detached by adding 2 mL of trypsin, then gently tapped to ensure complete dissociation. Fresh DMEM was used to terminate trypsinization, and the cell suspension was reseeded at a 1:10 ratio by transferring 1 mL into a new 75 cm^2^ flask with fresh medium. For experiments, cell counts were obtained using a Bio-Rad automated cell counter with Trypan blue staining. The inhibitor of ISR signaling ISRIB was administered at a concentration of 450 µM for 72 h. 3-AT and Setanaxib were used at concentrations of 10 mM and 1 µM for 24 h, respectively. MEFs were transfected with gene-targeting siRNA using Lipofectamine RNAiMAX (Invitrogen). Depending on the downstream assay, single or double transfections were performed, with scrambled siRNA serving as a control. Cells were processed 48 h post-transfection. Viability was assessed using the Trypan Blue Exclusion Assay. All cell fractions (medium, PBS washes, and detached cells) were collected, centrifuged at 300 g for 5 min, and resuspended in fresh medium. A 1:1 mixture of cell suspension and 0.4% Trypan Blue was prepared, incubated for 1–2 min, and analyzed with an automated cell counter.

### Electron microscopy

After sacrificing, the mice were perfused with a fixative solution consisting of 4% paraformaldehyde, 1,5% glutardialdehyde, and 2% sucrose in 0,1 M sodium cacodylate buffer at pH 7.3 [51, 52]. Subsequently, the organs were cut into blocks of 2mm^2^ and immersed in the fixation solution. The tissue blocks were immersed in 1% glutardialdehyde in 0,1 M sodium cacodylate buffer (pH 7.3) for a period of five minutes. Subsequently, the tissue was immersed in 0.1 M sodium cacodylate buffer at pH 7.3 for a total of two 5-min intervals, followed by a 5-min immersion in 0,01 M Teorell Stenhagen (TS) buffer at pH 10.5 repeated five times. The diaminobenzidine (DAB) reaction was conducted for a period of two hours at a temperature of 45 °C. The tissue was then immersed in a solution of 0,2% DAB and 0,15% H₂O₂ in TS buffer at pH 10.5. Subsequently, the tissue was immersed in 0,01 M TS buffer (pH 10.5) for a period of five minutes and in 0,1 M sodium cacodylate buffer (pH 7.4) for a period of three 10-min intervals at room temperature. Subsequently, the tissue was immersed in 2% osmium tetroxide (OsO4) in 0.1 M sodium cacodylate buffer pH 7,4 for one hour, after which it was immersed in 0,1 M sodium cacodylate buffer pH 7.4 for four 5-min intervals and washed in aqua dest. for three 5-min intervals. Subsequently, the tissue was immersed in 4% uranyl acetate in distilled water for a period of 24 h at room temperature. On the following day, the tissue was washed in aqua dest. for a total of three 5-min intervals and then embedded in epoxy resin (EPON). The tissue was immersed in ascending acetone solutions for 10 min each at a series of concentrations, beginning with 70% acetone in aqua dest., followed by 80%, 90% and 100% acetone. Subsequently, the tissue was immersed in a 1:1 solution of acetone and EPON for a period of two hours, followed by immersion in pure EPON overnight. On the following day, the tissue was embedded in EPON and allowed to polymerise for 48 h at a temperature of 60 °C. The tissue sections were cut to a thickness of 60–70 nm with an Ultracut E microtome (Reichert-Jung, Nußloch, Germany). The sections were scanned with a transmission electron microscope (LEO 912 AB) using the Image SP(64) software. The number of peroxisomes was manually counted. The size of the peroxisomes was analyzed using the ImageJ software. The area of peroxisomes was quantified by manually delineating the peroxisome boundaries and utilizing an automated measurement process. To calculate the area proportion of peroxisomes, the total cardiomyocyte area was measured by subtracting the area of vessels and artefacts.

### Gene expression analysis

Total RNA was extracted using the NEB Monarch RNA Isolation Kit (New England BioLabs) following the manufacturer's protocol and treated with Rnase-free DNase I (Thermo Fisher Scientific) to remove residual DNA. cDNA was synthesized from 100 ng of RNA using a first-strand cDNA Synthesis kit (Thermo Fisher Scientific) according to the manufacturer’s protocol. RNA and cDNA quality were assessed using a NanoDrop™ spectrophotometer. Gene expression levels were determined using quantitative real-time PCR (qPCR) with SensiMix SYBR Lo-ROX (Bioline) and analyzed with the RealTime System (C1000 Touch Thermal Cycler, Bio-rad). Gene expression was normalized to GAPDH or B-actin and analyzed using the ΔΔCt method. The primers used are indicated in the tables below.

**Primer sequences**

**Table:** 

Target genes	Primer sequence 5´-3´	
*Atf4*	forward	GCCGGTTTAAGTTGTGTGCT
	reverse	CTGGATTCGAGGAATGTGCT
*Chop*	forward	TATCTCATCCCCAGGAAACG
	reverse	GGGCACTGACCACTCTGTTT
*Gadd45*	forward	GCTCAACGTAGACCCCGATA
	reverse	GTTCGTCACCAGCACACAGT
*Gapdh*	forward	CATCACTGCCACCCAGAAGACTG
	reverse	ATGCCAGTGAGCTTCCCGTTCAG
*Mthfd2*	forward	CAGAGGAGCTGGAAGTGTTCAA
	reverse	TGGCTCAGAGTGCTGCTAGTTG
*Psat1*	forward	CAGGTCAGTGGGAGGCATTC
	reverse	GGCCGCCAGCTTCTCAA
*Gnpat*	forward	CTCCTGGTGGTCACCATTCT
	reverse	GCCAGGTTAGAGTGCAGGAG
*Catalase*	forward	TGGGACCCAACTATCTGCAG
	reverse	TGTGTAGAATGTCCGCACCT
*Nqo1*	forward	GGTAGCGGCTCCATGTACTC
	reverse	TGCCCTGAGGCTCCTAATCT
*B-actin*	forward	GGCTGTATTCCCCTCCATCG
	reverse	CCAGTTGGTAACAATGCCATGT
*Hmox1*	forward	GGAAATCATCCCTTGCACGC
	reverse	CTAGCAGGCCTCTGACGAAG
*Puma (Bbc3)*	forward	GTCTAGCCCGCGACAGTC
	reverse	AGTTGGGCTCCATTTCTGGG
*Bim (Bcl2l11)*	forward	TGGCCAAGCAACCTTCTGAT
	reverse	GCCTTCTCCATACCAGACGG
*Rplp0*	forward	TCACTGTGCCAGCTCAGAAC
	reverse	ATCAGCTGCACATCACTCAGA
*Far1*	forward	CCTTGCACAGCAAATGAAGA
	reverse	CCTTCCTGCTGCACAACATA
*Maoa*	forward	CACCGGTTTTGACTGCCAAG
	reverse	GCACCTTCCATGTAGCCACT
*Maob*	forward	ATCTCTCGTGTGCCTTTGGG
	reverse	AGATTCTGGTTCTGGCTGCC
*Nox2*	forward	TTTGTCAAGTGCCCCAAGGT
	reverse	TGCACAGCAAAGTGATTGGC
*Nos2*	forward	TTCACCCAGTTGTGCATCGACCTA
	reverse	TCCATGGTCACCTCCAACACAAGA

**siRNA sequences**

**Table:** 

Target gene	siRNA sequences	Supplier
siGNPAT	5'-CCA UUC GGU UCU UUG CCU UUU TT-3'	Microsynth, Balgach (Switzerland)
siCAT	5'-CCA GAU ACU CCA AGC CAA ATT-3'	Microsynth, Balgach (Switzerland)
Negative control (scrRNA)	5'-AGG UAG UGU AAU CGC CUU G-3'	Microsynth, Balgach (Switzerland)

**Antibodies**

**Table:** 

Name	Supplier	Identifier
ABCD3	Abcam	ab3421
Beta-Tubulin	Abcam	ab6046
ATF4	Cell Signal	11,815
Glyceraldehyd-3-phosphat-dehydrogenase antibody (GAPDH)	Millipore	374
Catalase	Abcam	1,935,058
Caspase 3	Cell signaling	9662
FAR1 (MLSTD2)	Abcepta	ABC-BP9396a
GNPAT	Proteintech	14,931–1-AP
MTHFD2 Polyclonal antibody	Proteintech	12,270–1-AP
PERK Antibody	Cell Signaling	3192
PEX14	ProteoGenix	22,173-A01
PEX19	Invitrogen	PA5-22,129
Phospho-PERK Antibody	Cell Signaling	3179
PHYH	Santa Cruz	sc-376727
PSAT1 Polyclonal antibody	Proteintech	10,501–1-AP

**Table:** 

Secondary Antibody	Host	Method	Dilution	Supplier
Anti Rabbit-IgG Alexa Fluor 488	Donkey	IF	1:600	Jackson Immono Research
Anti mouse IgG alkaline phosphatase	Goat	WB	1:10,000	Jackson Immono Research
Anti rabbit IgG alkaline phosphatase	Goat	WB	1:10,000	Jackson Immono Research
Alexa mouse 488	Mouse	IF	1:1000	Life Tech A-11001
Alexa rb 647	Goat	IF	1:1000	Life Tech A-21244
Alexa Flour rb 594	Goat	IF	1:1000	Life Tech A11012

### Protein extraction and quantification

Whole-cell and tissue lysates were prepared in RIPA buffer (50 mM Tris–HCl pH 7.4, 1% NP-40, 0.5% Na-deoxycholate, 0.1% SDS, 150 mM NaCl, 2 mM EDTA, 50 mM NaF), supplemented with 0.5 mM PMSF, complete protease inhibitors, and phosphatase inhibitors. Tissue samples were homogenized in RIPA buffer using eppi-pestles on ice. After a 5-min incubation on ice, the samples were vortexed for 30 s at maximum speed and centrifuged at 3000 rpm for 10 min at 4 °C. The supernatant was collected for analysis. Protein concentration was determined using a Bradford assay with a standard curve generated from IgG protein, and absorbance was measured at 750 nm.

### SDS-PAGE and western blotting

Proteins were separated in SDS-PAGE and transferred to methanol-activated PVDF membranes in a semi-dry blotting technique (230 mA, 2 h). Protein concentrations were measured with the Bradford assay to ensure equal loading. Membranes were blocked with 5% milk in TBST (200 mM Tris/HCl (pH 7.4), 1.25 M NaCl, 0.1% Tween 20), incubated with primary antibodies for 1 h at room temperature or overnight at 4 °C, and washed three times for 10 min with TBST. Detailed information to the antibodies can be found in the supplementary antibody table. Membranes were then incubated with secondary antibodies for 1 h, followed by additional washes. Proteins detected with ECL and imaged with a ChemiDoc™ Imager. Signal intensities were determined with Image Lab 6.0. To ensure direct comparison, protein samples were analyzed on the same gel to stain proteins on the same membrane. The proteins of similar size were decorated in a sequential manner, with the weakest antibodies first. The membranes were then washed and then redecorated with the stronger control antibody.

### Catalase activity assay

The catalase activity was determined by measuring hydrogen peroxide (H₂O₂) decomposition. The stop solution consisted of 22.47 mM titanium (IV) oxysulfate (TiOSO₄) in 2 M sulfuric acid (H₂SO₄). A standard curve was generated using H₂O₂ concentrations from 0 to 20 mM. Cell lysates were prepared in 0.33 M sodium phosphate-buffered (pH 7.0) with 1% Triton X-100. A fresh H₂O₂ working solution was prepared from a 30% stock. For the reaction mix, 41.25 mM sodium phosphate buffer (SPB), 9.36 mM TiOSO₄, and 20 mM H₂O₂, with added distilled water or with added sample lysate, according to the standard curve protocol, were prepared. Samples were incubated for 3 min at room temperature; afterward, 9.36 mM TiOSO₄ was added, and absorbance was measured at 410 nm. Catalase activity was defined as the amount of H₂O₂ degraded by a milligram of protein and calculated by linear regression from the standard curve.

### Immunofluorescence and oxidative stress assays

MEFs were grown on coverslips to analyze protein localization and oxidative stress, then fixed in 4% PFA for 15 min at room temperature, followed by PBS washes, pH 7.4. Cells were permeabilized with 1% glycine in PBS for 10 min and then washed three times with PBS containing 0.05% Tween 20. Cells were then incubated with 0.1% Triton X-100 for 5 min to ensure complete membrane permeability, followed by additional washes. Blocking was performed with 5% BSA in PBS for one hour at room temperature. Immunofluorescence staining, primary antibodies (1:1000 in PBS) were incubated overnight at 4 °C in a humidified chamber. The next day, coverslips were washed and incubated with Alexa Fluor-conjugated secondary antibodies (1:500 in PBS, Invitrogen) for 1 h in the dark. Nuclei were counterstained with DAPI (1:5000 in PBS) for 10 min, followed by washes. Coverslips were mounted using Mowiol and stored overnight at 4 °C before imaging. The extent of oxidative stress was assessed by staining cells with CellROX™ Deep Red (Invitrogen) at 5 µM for 30 min at 37 °C, followed by PBS washes and fixation. For measuring lipid peroxidation, BODIPY 581/591 C11 (Cayman Chemical) was used: a lipophilic dye that shifts its fluorescence from red (590 nm) to green (510 nm) upon oxidation. Both dyes have been incubated under light-protected conditions using the same staining profile. Imaging was performed by confocal microscopy at specific excitation/emission wavelengths for CellROX™ Deep Red (640/665 nm) and BODIPY 581/591 C11 (488 nm excitation; 510 nm oxidized, 590 nm non-oxidized). The cells were first imaged on an immunofluorescence microscope for quality control of the staining. High-resolution confocal imaging was performed with a 60 × objective, and fluorescence intensity was quantified using Fiji (ImageJ). Comparative analysis of cytosolic and mitochondrial ROS was measured via live cell staining, using 5 µM CellROX™ Deep Red (Invitrogen) or 5 µM MitoSOX™ red. Imaging was performed by confocal microscopy at specific excitation/emission wavelengths for CellROX™ Deep Red as before and MitoSOX™ red (510/580 nm) in a humidified and temperature controlled (37 °C) environment.

### Statistical analysis

Data were analyzed using GraphPad Prism. Student’s t-tests or ANOVA with post-hoc tests were applied as appropriate. P-values < 0.05 were considered statistically significant. Detailed statistical methods are provided in the figure legends.

## Supplementary Information

Below is the link to the electronic supplementary material.

Supplementary file1 (DOCX 634 KB)

## Abbreviations

1C: One carbon metabolism
BTHS: Barth Syndrome
CL: Cardiolipin
ER: Endoplasmic reticulum
FAO: Fatty acid oxidation
GSH: Glutathione
ISR: Integrated stress response
PCR: Polymerase chain reaction
ROS: Reactive oxygen species
shRNA: Short-hairpin-RNA
siRNA: Small interfering RNA
*Taz-*KD: *Tafazzin*-Knockdown
*Taz*^KO^: *Tafazzin*-Knockout
WT: Wild type
