# Supplementary material for: Peroxisomal catalase and plasmalogen biosynthesis protect from oxidative stress in Barth syndrome cardiomyopathy
Source: Basic Res Cardiol. 2026 Jun 30;121(5):1077–102. doi: 10.1007/s00395-026-01197-2 (PMC13593645; doi:10.1007/s00395-026-01197-2)
Supplement: Supplementary file 1 [file 395_2026_1197_MOESM1_ESM.docx]

**Supplementary Figure 1: (A)** qPCR of *Taz* gene expression in *Taz*-KD versus WT control in 10-week (left panel) and 50-week-old mice (right panel). Values are presented as mean ± SEM, WT 10 weeks n=5, *Taz*-KD 10 weeks n=6, both groups 50 weeks n= 4, statistical significance was determined with unpaired Student’s t-test. **(B)** DAB-stained electron micrographs of 50-week-old WT and *Taz*-KD peroxisomes. Quantification of 113 (WT) and 191 (*Taz*-KD) DAB-stained electron micrographs at 50 weeks of age, n=3. **(C)** Analysis of gene expression via qPCR for the indicated genes in 10-week-old *Taz*-KD hearts. Values are presented as mean ± SD, P-value: *P < 0.05, **P < 0.01. *Taz*-KD 50-week-old n=3, further groups n=4, statistical significance was determined with unpaired Student’s t-test. **(D)** Cardiac lysates of 10 week old mice were subjected to western blot analysis using antibodies against PEX14, PEX19 and LONP1. Protein amounts were normalized to Coomassie staining of whole proteins (values are presented as mean ± SD, n=3). **(E)** Representative double stained mouse heart section. ABCD3, green, PEX14, red, DAPI, blue. Black dots in the right panel represent colocalized areas. Scale bars: 50 µm (overview), 10 µm (inset). **(F)** Western blot analysis of PEX14 and GAPDH as a control in 50-week-old *Taz*-KD hearts. Quantification of PEX14, normalized to GAPDH in four animals in the right panel (values are presented as mean ± SD, n=4). **(G)** Western blot analysis of PEX14 and PEX5, normalized to GAPDH in 30-week-old *Taz*-KD livers. Quantification of particles in the right panel (values are presented as mean ± SD, n=3).

**Supplementary Figure 2: (A)** Quantification of gene expression of peroxisomal FAO enzymes in 10-week-old (upper panels) and 50-weeks old (lower panels) *Taz-*KD hearts. Values are presented as mean ± SD, *Taz*-KD 50-week-old n=3, further groups n=4. **(B)** Representative ABCD3 (red) staining in 10-week-old WT and *Taz*-KD mouse hearts, nuclear staining with DAPI in blue. **(C)** Quantification of ABCD3 particle count (left panel) and particle intensity (right panel) Values are presented as mean ± SD, n=3. **(D)** Western blot quantification of .cardiac lysates (50 weeks) and normalization of ACAA1 to TUBULIN as a control. **(E)** Quantification of western blot analysis of cardiac lysates (50 weeks) and normalization of PHYH to TUBULIN as a control. **(F)** Western blot analysis of ABCD3 and PHYH in 30-week-old *Taz*-KD liver, normalized to GAPDH. ACAA1 was normalized to TUBULIN as a control. Quantifications are presented as mean ± SD, n=3. Statistical significance was determined with unpaired Student’s t-test).

**Supplementary Figure 3: (A)** Analysis of catalase protein levels in 50 week old heart tissue. Quantification of catalase, normalized to GAPDH in the right panel (values are presented as mean ± SD, n=3). **(B)** Western blot analysis of catalase and GAPDH in 50 week old kidney, n=3. Quantification in the right panel (values are presented as mean ± SD, n=3) **(C)** Western blot analysis of catalase and GAPDH in 50 week old adipose tissue, Quantification in the right panel (values are presented as mean ± SD, n=3) **(D)** Quantification of catalase levels in 50 week old liver (values are presented as mean ± SD, n=3)**. (E)** Western blot analysis of catalase in MEFs, treated with 10 mM of 3-AT. Right panel shows quantification of three independent experiments. Values are presented as mean ± SD. Statistical significance was determined two-way ANOVA. **(F)** Catalase activity in MEFs, treated with 3-AT and left untreated. Values are presented as mean ± SD, n=3 statistical significance was determined with unpaired Student’s t-test.

**Supplementary Figure 4: (A)** Immunofluorescence using CellRox (red, upper panel) and MitoSOX (red, lower panel) in MEF treated with 10 mM 3-AT or left untreated. Nuclei stained with Hoechst 33258 (blue). Oxidation of MitoSOX by 10 µM of the mitochondrial targeted redox-cycler Mito-Paraquat (Mito-PQ) was used as an internal control (data not shown) **(B)** qPCR analysis of expression of indicated genes in *Taz*^KO^ MEFs. Values are presented as mean ± SD, n=3 statistical significance was determined with unpaired Student’s t-test. **(C)** CellRox fluorescence in MEF treated with 10 mM 3-AT and 1 µM Setanaxib (Setan). Nuclei stained with DAPI (blue). **(D)** CellRox fluorescence in MEF treated with 10 mM 3-AT or 150 µM BSO or a combination of both. Nuclei stained with DAPI (blue).

**Supplementary Figure 5: (A)** qPCR analysis of gene expression, involved in plasmalogen biosynthesis in 10-week-old *Taz*-KD hearts. Values are presented as mean ± SD, n=3 statistical significance was determined with unpaired Student’s t-test. **(B)** Western blot analysis of FAR1 and GAPDH in *Taz*^KO^ MEFs upon siRNA-mediated knockdown of catalase. **(C)** Quantification of n=3 experiments shown in (B), values are presented as mean ± SD. **(D)** Western blot analysis of indicated proteins in 50-week-old *Taz*-KD livers with quantifications below. Values are presented as mean ± SD, n=5. Statistical significance was determined with unpaired Student’s t-test.

**Supplementary Figure 6: (A)** Western blot analysis for indicated proteins of cell lysates from scr and si*Gnpat* transfected WT and *Taz*^KO^ MEF. Gene expression analysis of indicated gene expression in MEF, transfected with siRNA against GNPAT or with non-targeting control. Analysis of gene expression of **(B)** *Gnpat* **(C)** *Chop* **(D)** *Psat* and **(E)** *Nqo1* by qPCR analysis. Values are presented as mean ± SD, n=4-5, statistical significance was determined two-way ANOVA followed by Tukey's multiple comparisons test. **(F)** Analysis of catalase gene expression, compared to Rplp0 as a control after administration of 20 µM Batylalcohol for 24h. n=4 **(G)** Western blot analysis of catalase and GAPDH as a control, after administration of 20 µM Batylalcohol for 24h. Values are presented as mean ± SD, n=3.
